# RNA Methylation in Cancer Metabolism: from Mechanisms to Therapeutic Opportunities

**DOI:** 10.7150/ijbs.124177

**Published:** 2026-01-01

**Authors:** Zeyu Wu, Yuncan Xing, Shiwen Mei, Tixian Xiao, Fangze Wei, Qian Liu

**Affiliations:** 1 Department of Colorectal Surgery, National Cancer Center/National Clinical Research Center for Cancer/Cancer Hospital, Chinese Academy of Medical Sciences and Peking Union Medical College, Beijing, China.; 2 Department of Clinical Laboratory, State Key Laboratory of Molecular Oncology, National Cancer Center/National Clinical Research Center for Cancer/Cancer Hospital, Chinese Academy of Medical Sciences and Peking Union Medical College, Beijing, China.

**Keywords:** RNA methylation, cancer, metabolism, clinical application

## Abstract

One of the most important changes in the transformation of normal cells into tumor cells is metabolism. In order to satisfy the more active proliferation, migration and metastasis of cancer cells, abnormal changes occur in various pathways and molecules involved in metabolism, which eventually lead to metabolic reprogramming of tumor cells. This process involves the uptake of nutrients and changes in major metabolic forms. As an important part of post-transcriptional epigenetics, RNA methylation modifications can regulate RNA processing and metabolism, while dynamically and reversibly influencing the expression of specific molecules, thereby ultimately affecting diverse biological processes and cellular phenotypes. In this review, various types of RNA methylation modifications involved in cancer are summarized. Subsequently, we systematically elucidate the mechanism of RNA modification for metabolic reprogramming in cancer, including glucose, lipid, amino acid and mitochondrial metabolism. Most importantly, we discuss in depth the clinical significance of RNA modification in metabolic targeted therapy and immunotherapy from mechanism to therapeutic application.

## 1. Introduction

RNA methylation modification first came into human view in the 1950s, when the first structurally modified nucleoside pseudouridine was labeled^1^. Over the following decades, more than 150 different classes of RNA modifications have been validated and discovered on cellular RNA^2^. However, researches on RNA modifications in disease have progressed slowly during this period. The past decade has seen a renaissance in RNA modification research, attracting increasing scientific attention^3^. In particular, in 2023, nucleoside base modification's contribution to the development of an mRNA vaccine against COVID-19 earned it a Nobel Prize that year, greatly inspiring biologists working on RNA-based therapies^4^. The landscape of post-transcriptional regulation is profoundly shaped by RNA modifications, among which RNA methylation stands out as one of the most abundant, reversible, and well-studied epigenetic mechanisms.

Cancer is one of the greatest threats to global public health. Despite significant progress in cancer detection and management, there are still tens of millions of new cancer cases and nearly half of cancer-related deaths occurring each year^5^, ^6^. A defining feature of cancer is metabolic reprogramming, an adaptive mechanism whereby cancer cells rewire their metabolic circuits to support rapid growth and enhance survival under stressful conditions. This metabolic shift enables the heightened energy generation necessary to fulfill the increased biosynthetic demands of proliferating cancer cells^7^. To achieve and sustain their proliferative advantage, cancer cells must activate or upregulate core metabolic pathways^8^. This phenomenon, characterized by dynamic alterations in metabolic patterns, encompasses several key areas: enhanced glycolysis, accelerated glutamine metabolism, upregulated lipid metabolism, modifications in amino acid metabolism, and mitochondrial adaptations. These metabolic changes are intricately shaped by the interplay between cancer cells and their surrounding tumor microenvironment^9^. Emerging evidence now underscores the role of RNA methylation as a pivotal regulator of this metabolic reprogramming. Functioning as a critical layer of post-transcriptional control, it allows cancer cells to swiftly adjust the expression and activity of metabolic enzymes and oncogenic signaling molecules in response to the fluctuating tumor microenvironment^10^.

This review synthesizes current knowledge on major RNA methylation modifications in cancer, with a dedicated focus on their interplay with metabolic reprogramming. Beyond delineating these specific regulatory roles, we critically assess the resulting therapeutic vulnerabilities and potential for targeting the RNA methylation machinery, concluding with a perspective on future research trajectories in this field.

## 2. The Writer, Reader, and Eraser Enzymes of RNA Methylation

### 2.1 m⁶A: The most prevalent RNA methylation modification

**Figure 1:**
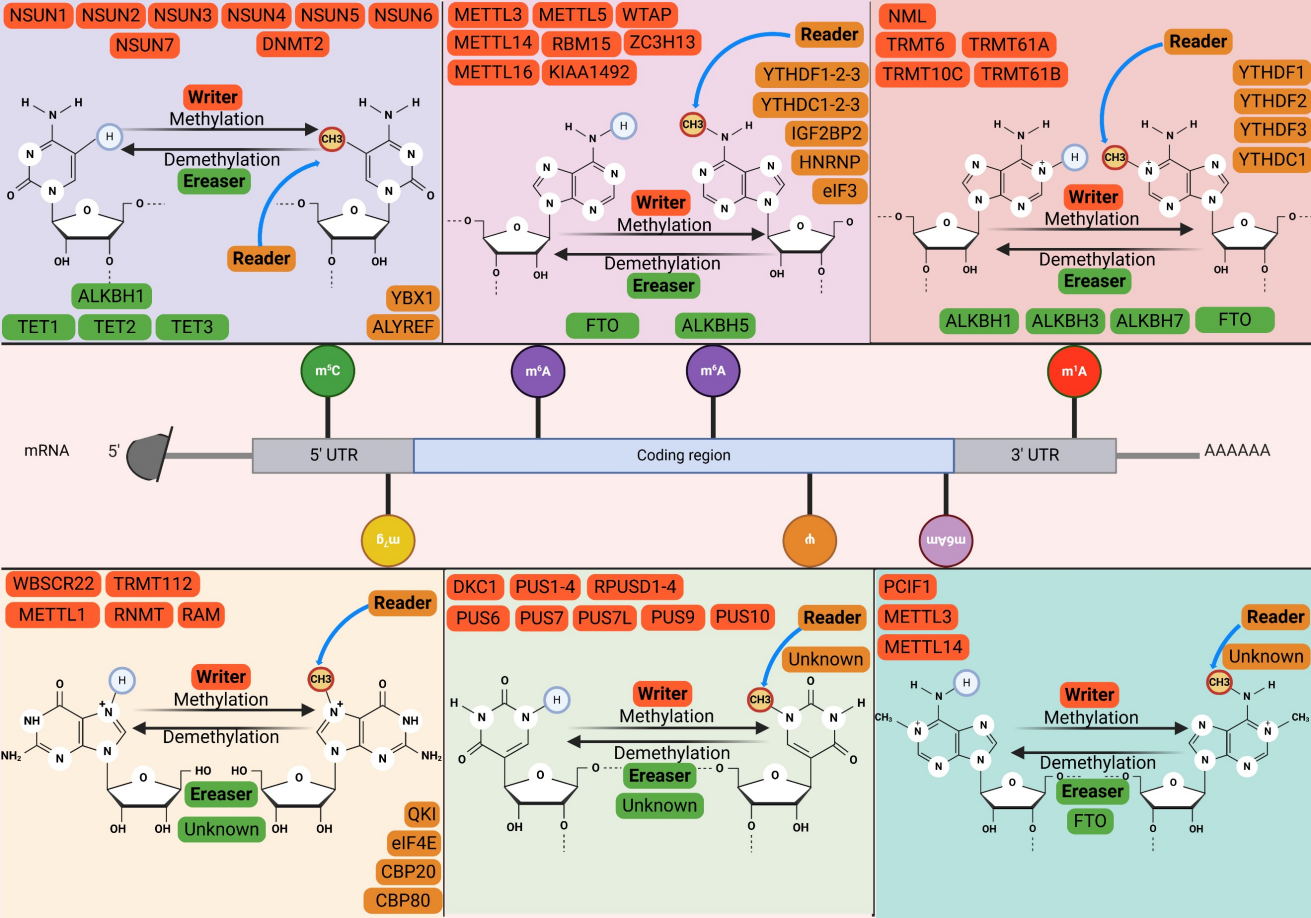
Overview of RNA methylation regulatory mechanisms. RNA methylation represents a dynamic and reversible epigenetic modification process. The major RNA methylation types include m⁶A, m¹A, m⁶Am, m⁷G and m⁵C. This modification process is precisely regulated by three functional protein groups: methyltransferases (writers, orange), demethylases (erasers, green), and recognition factors (readers, yellow), which collectively maintain the homeostasis of RNA methylation. m⁶A methylation: m⁶A methyltransferases catalyze the transfer of methyl groups to N6-adenosine residues on RNA. Conversely, demethylases, including FTO and ALKBH5, dynamically remove m⁶A modification sites from RNA, thereby attenuating the effects of methylation to varying degrees. Furthermore, m⁶A readers specifically recognize and bind to m⁶A-modified nucleotides, thereby activating or inhibiting downstream regulatory pathways. m⁵C methylation: m⁵C is catalyzed by writer proteins, primarily DNMT2 and members of the NSUN family, which deposit m⁵C modifications across diverse RNA transcripts. The removal of m⁵C methylation is mediated by eraser proteins, including TET1-3 and ALKBH1, which facilitate its demethylation. These dynamically regulated m⁵C modifications are recognized by reader proteins such as YBX1 and ALYREF, which subsequently influence RNA processing, stability, and nuclear export. m¹A Methylation: m¹A methylation is catalyzed by distinct methyltransferase complexes with specific substrate preferences. The TRMT6/61A complex recognizes a GUUCRA tRNA-like motif and promotes m¹A methylation at specific sites within mRNAs. TRMT61B mediates m¹A modification on mitochondrial mRNA transcripts, while TRMT10C methylates the A9 position of mitochondrial tRNALys and the m¹A site at position 1374 in ND5 mt-mRNA. The functions of demethylases and reader proteins in m¹A methylation are analogous to those characterized in the m⁶A methylation system. m⁶Am Methylation: PCIF1 specifically recognizes the 5'cap structure of mRNA and exhibits m⁶Am methyltransferase activity. METTL4 is capable of catalyzing m⁶Am methylation at specific sites in U2 snRNA and regulates pre-mRNA splicing. In terms of eraser, m⁶Am is preferentially and specifically demethylated only by FTO. To date, no dedicated reader proteins for m⁶Am have been reported. m⁷G Methylation: The METTL1/WDR4 complex primarily targets internal sites of mRNAs, the G46 position of tRNAs, and G-quadruplex structures within miRNAs. Meanwhile, the WBSCR22/TRMT112 complex predominantly catalyzes m⁷G modification on 18S rRNA, thereby facilitating its maturation. The RNMT and its activator RAM are responsible for the m⁷G modification at the mRNA 5' cap, which subsequently mediates nuclear export and translation initiation of mRNAs.

N6-methyladenosine (m⁶A), a classical and reversible RNA modification, is dynamically regulated by methyltransferases (“writers”) that install the mark and demethylases (“erasers”) that remove it^11^. The functional readout is executed by “reader” proteins, which recognize m⁶A and regulate downstream pathways to implement specific biological functions^12^. The mechanism of the main RNA modifications is illustrated in Fig. 1. m⁶A modification is generated through the co-modification of the methyltransferase complex, which comprises METTL3, METTL14, WTAP, VIRMA, ZC3H13, and RBM15/RBM15B^13^. The METTL3 domain, which exhibits an affinity for S-adenosylmethionine (SAM), transfers activated methyl groups to adenosine residues. METTL14 functions as an RNA-binding platform that facilitates the catalytic activity of METTL3^14^. Other writers, such as METTL16, ZCCHC4, and METTL5, are reported to facilitate the m⁶A modification of small nuclear RNA (snRNA), 28S ribosomal RNA (rRNA), and 18S rRNA, respectively^15^. Additionally, fat mass and obesity-associated protein (FTO) and alpha-ketoglutarate-dependent dioxygenase alkB homolog 5 (ALKBH5), which are dioxygenases, depend on Fe (II)/α-ketoglutarate and demethylate adenosine residues that have undergone modification via m⁶A methylation^16^. RNA-binding proteins (readers) can detect and adhere to m⁶A modification sites, regulating the function and structural composition of m⁶A -modified RNAs through diverse mechanisms^17^. Readers comprise various protein families, such as the YT521-B homology domain family (YTHDF), insulin-like growth factor 2 mRNA-binding proteins (IGF2BPs), and heterogeneous nuclear ribonucleoproteins (HNRNPs)^18^. In contrast to YTH family proteins, IGF2BP1, IGF2BP2, and IGF2BP3 exhibit comparable functions (enhancing mRNA stability and translation)^19^. The m⁶A modification is dynamically regulated by writers that install the mark, erasers that remove it, and readers that interpret it. Specifically, the deposited m⁶A marks are recognized by distinct reader proteins, such as YTHDF1, which promotes the translation of the modified mRNA. In contrast, recognition by YTHDF2 facilitates mRNA decay, thereby collectively enabling dynamic post-transcriptional gene regulation^13^.

### 2.2 m⁵C: The most abundant RNA methylation in eukaryotic transfer RNAs (tRNAs) and rRNAs

5-methylcytidine (m⁵C) is also a prevalent RNA alteration in different types of RNAs, such as cytoplasmic and mitochondrial rRNA and tRNA, mRNA, enhancer RNA (eRNA), and several non-coding RNAs^20^. m⁵C modification is catalyzed by the NOL1/NOP2/SUN domain (NSUN) protein family, which comprises seven distinct members (NSUN1-7)^21^. NSUN2, which is the most extensively investigated writer among the members of the NSUN family, mediates the introduction of m⁵C modifications into various RNAs, such as tRNA, microRNA (miRNA), long non-coding RNA (lncRNA), and mRNA^22^. Although NSUN1 and NSUN5 are localized within the nucleolus, they can alter the m⁵C modification of the 28S rRNA within the cytoplasmic milieu^23^. Nakano *et al.* demonstrated that the methylase NSUN3 initiates m⁵C modification in the mitochondrial tRNA of humans^24^. NSUN4, which is located in the mitochondria, serves as a multifunctional mitochondrial protein that facilitates the methylation of 12S rRNA and promotes the assembly of mitoribosomes^25^. Liu *et al.* structurally characterized NSUN6 in its apo form and after forming a complex with a full-length tRNA substrate. Furthermore, NSUN6 functions as a methyltransferase with specificity toward mRNA^26^. Selmi *et al.* reported that NSUN7 mediates the incorporation of m⁵C modifications into eRNA^27^. In contrast to m⁵C writers, limited studies have examined the erasers and readers of m⁵C modification. Previous studies have reported that ten-eleven translocation proteins (TETs) are m⁵C demethylases for DNA and mediate the conversion of m⁵C modification^28^. ALKBH1, a well-known eraser, can convert m⁵C modification into two modified forms at position 34 of both cytoplasmic and mitochondrial tRNAs^29^. The RNA-binding protein ALY/REF export factor (ALYREF) is a selective reader of m⁵C. ALYREF exhibits a specific affinity for the 5' and 3' regions of mRNA and regulates the transportation of mRNA from the nucleus^30^. Y-box binding protein 1 (YBX1), which is localized to the cytoplasm, functions as a reader that augments the stability of m⁵C-modified mRNAs^31^. The m⁵C modification is installed by writers (e.g., NSUN2) and interpreted by readers to direct diverse molecular outcomes. In mRNA, ALYREF recognizes m⁵C to promote nuclear export, facilitating protein synthesis. Whereas on tRNA, m⁵C deposition by NSUN2 and DNMT2 safeguards structural integrity and prevents degradation, thereby guaranteeing translational accuracy. This regulatory system is dynamically antagonized by erasers like TET proteins, which catalyze the reversal of m⁵C marks^32^.

### 2.3 m¹A: Associated with m⁶A

N1-methyladenosine methylation (m¹A) modification involves the addition of a methyl group to the first nitrogen atom of adenosine within RNA and was initially considered a major methylation modification for tRNA and rRNA^33^. In contrast to the distribution of m⁶A, m¹A is predominantly localized within the initiation codon and the 5' UTRs^34^. Additionally, m¹A is correlated with m⁶A, which can be partly attributed to the conversion of m¹A to m⁶A through Dimroth rearrangement under alkaline conditions^35^. The human tRNA m¹A methyltransferase, which is commonly referred to as the tRNA methyltransferase 6-tRNA methyltransferase 61A (TRMT6-TRMT61A) complex^36^. The complex serves distinct roles across RNA species. In tRNA, it safeguards structural integrity to ensure translational fidelity and efficiency. Within mRNA, the same mark actively promotes ribosomal translocation during translational elongation^37^. The enzymatic activity of tRNA methyltransferase 10C (TRMT10C) in conjunction with its binding partner protein Short-chain dehydrogenase/reductase family 5C member 1 (SDR5C1) promotes the introduction of m¹A into mitochondrial tRNA^38^. Zhang *et al.* demonstrated that ALKBH7 demethylates mitochondrial pre-tRNA. ALKBH1, ALKBH3, and FTO are reported to facilitate the demethylation of tRNA^39^, ^40^. Additionally, YTHDC1, but not YTHDC2, directly binds to m¹A modification in RNA^41^. However, further studies are needed to elucidate the functional role of YTHDC1.

### 2.4 m⁷G: Key after-cap regulator

N7-methylguanosine (m⁷G) is a highly conserved RNA modification widely present in both eukaryotes and prokaryotes^42^. The most well-known function of m⁷G modification is its role as the core component of the 5' cap structure (m⁷GpppN) of eukaryotic mRNA^43^. This 'cap' is essential for mRNA stability, nucleocytoplasmic transport, and the initiation of protein translation. However, with advances in high-throughput sequencing technologies, scientists have discovered that m⁷G modification is not confined to the 5' end of mRNA. It is also present internally in various RNA types, including tRNA, rRNA, miRNA, and within internal regions of mRNA^44^. The processes of writing, erasing, and reading m⁷G modifications are precisely regulated by specific enzymatic machinery^45^. The METTL1/WDR4 complex catalyzes m⁷G modifications in a large number of tRNAs and internal mRNA sites, where METTL1 serves as the catalytic subunit and WD repeat domain 4 (WDR4) is an essential auxiliary subunit for its stability and localization^46^. Catalysis is executed by RNA guanine-7 methyltransferase (RNMT) for the mRNA 5' cap during maturation^47^, by the METTL1-WDR4 complex internally in tRNAs/miRNAs to prevent cleavage and ensure stability, and by the Williams-Beuren syndrome chromosome region 22 (WBSCR22)/tRNA methyltransferase 112 (TRMT112) complex at specific 18S rRNA sites for ribosomal 40S subunit biogenesis^48^. Although irreversible due to the lack of known erasers, the m⁷G signal is interpreted by readers, exemplified by eukaryotic initiation factor 4E (eIF4E), which binds the mark to regulate downstream processes including mRNA translation and metabolism^49^, ^50^.

## 3. Dynamic Regulation of Cancer Metabolism by RNA Methylation

**Figure 2:**
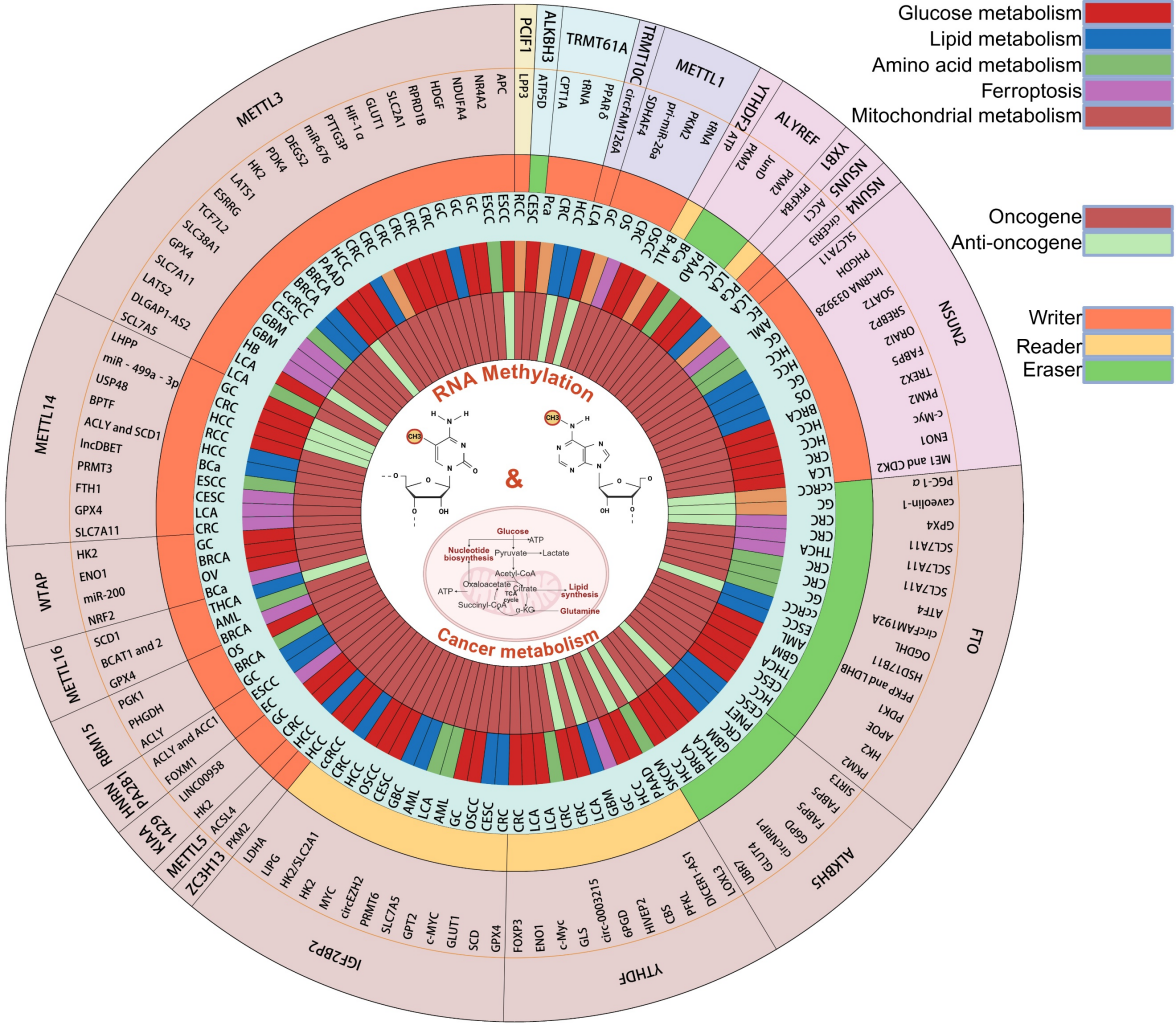
Roles of RNA methylation and downstream targets in cancer metabolism. The RNA methylation modifications can be classified into the following types: m⁶A, m⁵C, m⁷G, m¹A, and m⁶Am. In m⁶A and m⁵C modifications, the enzymes currently known to be associated with cancer metabolism include writers, readers, and erasers, which are represented by orange, yellow, and green colors respectively. For m⁷G and m⁶Am modifications, only writers have been identified to participate in cancer metabolism. Regarding m¹A modification, both writers and erasers are involved in cancer metabolism. The innermost ring displays downstream targets of RNA methylation in cancer, where tumor suppressor genes are shown in green and oncogenes in red. The second outer ring illustrates the metabolic pathways associated with RNA methylation, including glucose metabolism (red), lipid metabolism (blue), amino acid metabolism (green), ferroptosis (purple), and mitochondrial metabolism (orange).

This section centers on the pivotal phenomenon of cancer metabolic reprogramming to systematically elucidate the mechanistic basis of RNA methylation in regulating diverse oncogenic processes (Fig. 2).

### 3.1 RNA modifications regulate glucose metabolism in different cancers

Substantial evidence has demonstrated the involvement of RNA methylation modifications in diverse human malignancies, including gastrointestinal, reproductive, and urinary system cancers. This chapter specifically highlights RNA methylation-mediated regulation of cancer-associated glucose metabolic reprogramming (Table 1).

#### 3.1.1 Role of m⁶A modification in glucose metabolism in digestive system tumors

**Figure 3:**
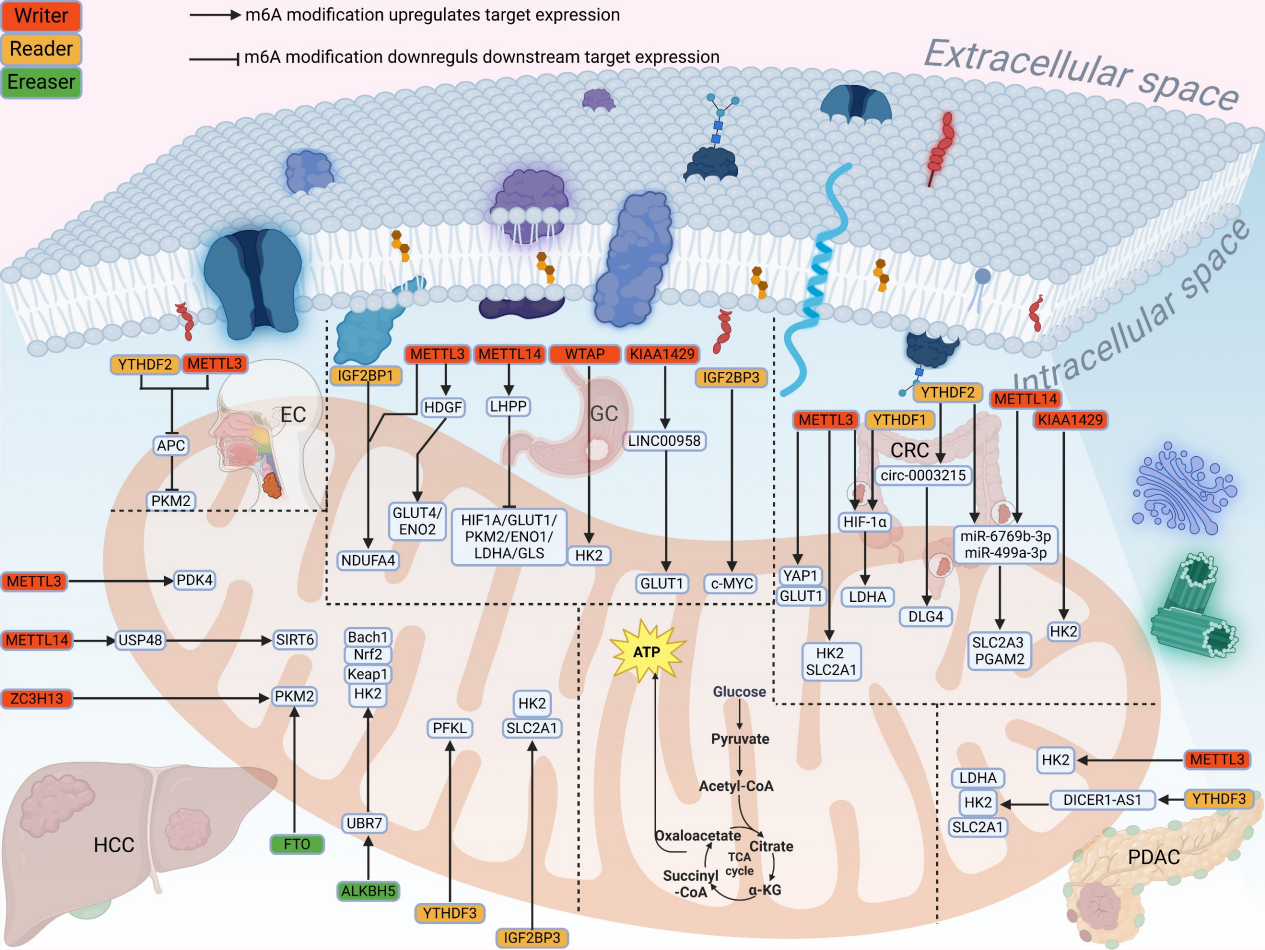
Mechanism of m⁶A methylation in metabolic reprogramming of digestive system tumors. The schematic illustrates the role of m⁶A methylation in metabolic reprogramming of digestive system tumors. Cancer cells predominantly rely on enhanced glycolytic pathways to efficiently generate ATP, meeting the high energy demands associated with tumor growth. This process is primarily achieved through altered tricarboxylic acid (TCA) cycle metabolism in mitochondria. The arrows indicate genes that promote glucose metabolism, and the blunted lines represent genes that inhibit glucose metabolism. In gastric, colorectal, hepatocellular, and pancreatic cancers, m⁶A writers (including METTL3, METTL14, WTAP, KIAA1429, and ZC3H13) augment m⁶A methylation on downstream target RNAs. Subsequently, m⁶A readers (including YTHDF1-3 and IGF2BP3) recognize the m⁶A modifications on glycolysis-related transcripts and facilitate their transcription, thereby enhancing glycolysis in tumor cells. In contrast, a distinct mechanism is observed in esophageal cancer, where METTL3 mediates m⁶A modification of APC mRNA, and YTHDF2 recognizes and promotes its decay, attenuating APC expression and consequently suppressing cancer progression.

In the following sections, we will describe the process by which m⁶A modifications are involved in glucose metabolism in digestive system tumors (Fig. 3). In esophageal cancer (EC), METTL3 was found to first increase the m⁶A modification of Adenomatous polyposis coli (APC) mRNA, and then recognized by reader YTHDF2 to reduce the expression of APC and promote the expression of β-catenin and Pyruvate kinase M2 (PKM2), thereby promoting glucose uptake and lactate production^51^. In esophageal squamous cell carcinoma (ESCC), WTAP mediates m⁶A modification on the lncRNA PDIA3P1, which is then recognized by IGF2BP1 to enhance its stability. Functionally, the stabilized PDIA3P1 acts as a competitive endogenous RNA for miR-152-3p, thereby preventing the degradation of Glucose transporter 1 (GLUT1) mRNA. Concurrently, it attenuates the interaction between MARCH8 and HK2, reducing HK2 ubiquitination and degradation. Consequently, these dual pathways synergistically enhance glycolysis, leading to increased lactate production and driving malignant progression^52^.

In gastric cancer (GC), Wang *et al.* found that METTL3 increased the m⁶A modification of HDGF mRNA to promote its expression, and IGF2BP3 further maintained the stability of HDGF mRNA, which in turn activated GLUT4 and ENO2 to enhance glycolysis^53^. Xu *et al.* confirmed that METTL3 could increase the m⁶A level of NDUFA4 mRNA, and IGF2BP1 further stabilized the expression of NDUFA4 to promote glucose uptake^54^. LHPP is affected by METTL14 to inhibit glycolysis-related proteins, thereby inhibiting aerobic glycolysis^55^. Further studies have shown that overexpression of WTAP enhances glucose uptake, lactate production, and extracellular acidification rate by promoting the stability of HK2 mRNA^56^. It was found that KIAA1429 promoted the remaining level of LINC00958 RNA in an m⁶A dependent manner. And the remaining level of GLUT1 mRNA was further increased, which promoted the aerobic glycolysis^57^. Part of LIN2B binds to c-MYC mRNA, the subsequent upregulated c-MYC increases glucose consumption and promotes glycolysis^58^. Shen *et al.* demonstrated that lactic acid production and glucose uptake were impaired to varying degrees after METTL3 knockdown. METTL3 regulated HK2 and SLC2A1, increasing the stability and expression of their mRNA^59^. METTL3 also regulates the GLUT1 to mediate glucose metabolism activated the mTORC1 signaling pathway^60^, triggers the translation of LDHA mRNA through methylation and recruitment of YTHDF1 to reduce its expression, thus affecting the glycolysis process^61^. Overexpressed LncRNA PTTG3P can promote the glycolysis. METTL3 can enhance the stability of PTTG3P and IGF2BP2 also binds to the m⁶A site to increase its expression^62^. YTHDF2 can bind to SLC2A3 and PGAM1, and thus be positively regulated by METTL14 to increase these two precursor RNAs to weaken glycolysis in p53-WT CRC^63^. KIAA1429 increases the m⁶A modification level of HK2 mRNA and thus promotes its expression, and accelerates glycolysis^64^. circ-0003215 reduces the expression of miR-663b by sponging it, thereby relieving its inhibition of the downstream target DLG4. YTHDF2 binds to circ-0003215 and reduces its m⁶A level leading to a decrease in its expression^65^. In addition, WTAP can activate m⁶A modification by mediating the binding of YTHDF1 to specific sites of FOXP3 mRNA, which further promotes glycolysis by activating the transcription of SMARCE1^66^. METTL3 promotes PDK4 expression and can effectively promote the glycolysis of cancer cells and increase the production of ATP in HCC^67^.

The loss of USP48 can significantly enhance the metabolic flux of aerobic glycolysis. METTL14 stabilizes the mRNA expression of USP48 through m⁶A modification, indirectly participating in this cancer inhibition^68^. ZC3H13 can significantly reduce the stability of PKM2 mRNA, thereby reducing glucose uptake and lactic acid production^69^. FTO knockdown can lead to upregulation of m⁶A modification level, thereby reducing PKM2 mRNA and protein levels and inhibiting aerobic glycolysis^70^. UBR7 can upregulate Keap1 and further inhibits Nrf2/Bach1 pathway, finally inhibits HK2 and glycolysis. Overexpression of ALKBH5 can stabilize the expression of UBR7 mRNA to affect glycolysis^71^. YTHDF3 can promote PFKL mRNA to promote glycolysis. Furthermore, PFKL protein inhibits the ubiquitination of YTHDF3 protein by EFTUD2, thus forming the positive feedback^72^. Ye *et al.* found that lncRNA miR4458HG can interact with IGF2BP2 to stabilize HK2 and SLC2A1 mRNA. SLC2A1 further encodes GLUT1 to promote the efficiency of glycolysis^73^. METTL3 binds to specific sites on AKR1B10 mRNA and enhances its m⁶A modification level, thereby upregulating AKR1B10 expression. Consequently, the elevated AKR1B10 promotes a glycolytic phenotype in cholangiocarcinoma (CCA) cells, characterized by increased glucose uptake and lactate production, which ultimately drives malignant progression^74^. Peripheral nerve invasion of the nerves within pancreatic ductal adenocarcinoma (PDAC) is one of the causes of early metastasis. Li *et al.* confirmed that neural cells can promote the expression of METTL3 and enhance the glycolytic capacity of PDAC cells by increasing m⁶A modification of HK2 mRNA^75^. lncRNA DICER1 antisense RNA DICER1-AS1 promotes the transcription of DICER1, which further promotes the expression of miR-5586-5p, and subsequently negatively regulated four glycolytic genes, thereby inhibiting the glycolysis. More importantly, YTHDF3 can reduce the stability of DICER1-AS1 from the source^76^.

#### 3.1.2 Role of m⁶A modification in glucose metabolism in non-digestive system tumors

High expression of YTHDF2 in lung cancer causes the specific methylation of 6-phosphogluconate dehydrogenase (6PGD) mRNA. The upregulation of 6PGD significantly activates the pentose phosphate pathway (PPP) and promotes the glucose metabolism process^77^. Upregulated METTL3 and downregulated ALKBH5 promote m⁶A modification by binding of ENO1 mRNA, while highly expressed ENO1 activates glycolysis then^78^. Another study found that FTO could significantly inhibit the expression of c-Myc, thus inhibiting the glycolysis. This process is negatively regulated by YTHDF1^79^. METTL3 increased stability of DLGAP1-AS2 by binding to its m⁶A specific site. Upregulated DLGAP1-AS2 promotes the expression of c-Myc to activate aerobic glycolysis in non-small cell lung cancer^80^. IGF2BP3 promotes glycolysis in oral squamous cell carcinoma (OSCC) cells by upregulating the expression of GLUT1^81^. IGF2BP2 was also found to stabilize HK2 mRNA, and the aberrant increase of HK2 promoted the glycolysis^82^. LATS1 can inhibit the glycolysis in breast cancer cells. Overexpression of METTL3 increased the m⁶A modification of LATS1 mRNA, and then YTHDF2 decreased the mRNA stability of LATS1 by recognizing its m⁶A site^83^. In addition, IL1β was found to synergize with TNFα in breast cancer cells to activate ERK1/2 to upregulate the expression of WTAP, which promotes the expression of ENO1 to promote the glycolysis^84^. It was also found that ALKBH5 promotes the demethylation of GLUT4, and decreases the binding of YTHDF2 to the m⁶A site of GLUT4. The upregulation of GLUT4 promotes glycolysis in HER2-targeting-resistant breast cancer cells^85^. Human papilloma virus (HPV) 16 E6/E7 can upregulate IGF2BP2 and recognize MYC mRNA to increase aerobic glycolysis^86^. In addition, FTO inhibits glycolysis in cervical cancer cells by down-regulating HK2 expression^87^. Under hypoxic conditions, hypoxia-inducible factor 1α (HIF-1α) upregulates the expression of WTAP, which in turn enhances the proliferation and invasion of ovarian cancer (OC) cells. Mechanistically, WTAP significantly increases m⁶A modification on pri-miR-200 and facilitates its processing into mature miR-200 by interacting with DGCR8. Furthermore, studies demonstrate that miR-200 upregulates the key glycolytic enzyme HK2, thereby significantly promoting the Warburg effect within cancer cells^88^. In thyroid cancer, knockdown of ALKBH5 significantly upregulates the expression of circNRIP1 and further upregulates PKM2 to promote glycolysis^89^. APOE, as a carcinogenic agent, promotes glycolysis through the IL-6/JAK2/STAT3 signaling pathway. This process is regulated by FTO to reduce the m⁶A modification level of APOE mRNA in thyroid cancer cells^90^. In gliomas, ALKBH5 stabilizes G6PD mRNA by demethylating m⁶A modification sites to activate the PPP^91^. lncRNA JPX increases the stability of PDK1 mRNA in an FTO-dependent manner, thereby promoting glycolysis, proliferation and TMZ resistance in glioblastomas^92^. R-2-hydroxyglutaric acid (R-2HG) effectively inhibits glycolysis in leukemia cells, and this metabolite is used to inhibit downstream targets PFKP and LDHB by inhibiting demethylation from FTO^93^. In osteosarcoma, RBM15 promotes glycolysis by upregulating the expression of three key metabolic enzymes, HK2, GPI, and PGK1^94^. In renal cancer, IGF2BP1 can increase the expression of LDHA and promote glycolysis^95^. Low expression of METTL14 can release the inhibition of BPTF and activate downstream targets ENO2 and SRC, promoting the glycolysis of RCC cells^96^. In Diffuse large B-cell lymphoma (DLBCL), piRNA-30473 enhances the stability of WTAP mRNA by binding to its 3' UTR, thereby reducing its decay. The upregulated WTAP, in turn, promotes the expression of HK2 by targeting its transcript's 5' UTR. Concurrently, another reader protein, IGF2BP2, exerts a similar promotional effect on HK2 expression. Collectively, this regulatory axis ultimately leads to increased glycolysis^97^.

#### 3.1.3 Roles of other RNA modifications in glucose metabolism in cancers

In recent years, many studies have focused on the metabolic reprogramming involved in m⁵C modification, especially in glucose metabolism. In TNBC, the majority of tRNA m⁵C modifications have a strong positive association with NSUN2 levels, with tRNA^Val-CAC^ exhibiting the most pronounced correlation. Functional assays show that overexpression of NSUN2 and tRNA^Val-CAC^ can significantly enhance glucose uptake, lactate production, and intracellular ATP levels, indicating a promotion of glycolytic metabolism^98^. In BCa cells, knockdown of YBX1 suppresses proliferation, migration, and invasion, while also attenuating glycolytic activity. Mechanistically, this occurs through YBX1-dependent m⁵C modification that enhances the stability of TM4SF1 mRNA. The upregulated TM4SF1 activates the β-catenin/c-Myc signaling pathway, ultimately promoting glycolysis^99^. It was found that ALYREF binds to the m⁵C specific site of PKM2 mRNA to increase its stability, thus promoting the glycolysis process^100^. Yu *et al.* initially found that THOC3 is capable of promoting glucose utilization rate, lactate production and intracellular ATP levels of LUCS cells. Mechanically, THOC3 exports PFKFB4 mRNA to the cytoplasm, and combines with YBX1 to stabilize its expression^101^. It was reported that NSUN2 significantly upregulated in the lung tissue of mice. It could increase the stability of ME1 and GLU3 mRNAs in an m⁵C dependent manner, resulting in an effect on metabolic reprogramming^102^. In GC, NSUN2 enhances the m⁵C modification on PGK1 mRNA, which is recognized by YBX1, leading to the upregulation of PGK1 and consequently promoting glycolysis. Furthermore, the NSUN2/PGK1 axis activates the PI3K/AKT signaling pathway, ultimately contributing to the development of malignant phenotypes^103^. Similarly, NSUN2 can increase m⁵C modification of ENO1 mRNA, while YBX1 recognizes and stabilizes it. ENO1 then promotes glucose metabolism in CRC cells^104^. In HCC, knockdown of NSUN2 significantly inhibits glycolytic genes such as ENO1, LDHA, PKM2 and TPI1, which is due to the m⁵C modification of c-Myc mRNA^105^. NSUN2 stabilizes PKM2 expression by elevating m⁵C methylation, thereby enhancing glycolytic flux in HCC cells^106^. In intrahepatic cholangiocarcinoma (ICC), ALYREF stabilizes PKM2 mRNA and subsequently promotes glycolytic metabolism^107^. Interestingly, it has been observed that glucose acts as a cofactor for NSUN2 by binding to its N-terminal domain (amino acids 1-28), thereby upregulating its enzymatic activity. The activated NSUN2 then stabilizes TREX2 mRNA, leading to increased TREX2 protein expression and the subsequent inhibition of interferon responses^108^. In RCC, downregulation of NSUN2 diminishes glycolytic capacity by reducing the RNA stability of ENO1. Conversely, NSUN2 overexpression enhances lactate production by upregulating ENO1. The accumulated lactate promotes histone H3K18 lactylation, which in turn upregulates PD-L1 expression via the TOM121/MYC/CD274 signaling axis, ultimately facilitating immune escape^109^. In OC, ALYREF directly interacts with BIRC5, and their expressions are positively correlated. Silencing ALYREF suppresses glycolysis, while the consequent antitumor effects can be rescued by BIRC5 overexpression, indicating BIRC5 functions downstream of ALYREF^110^. In retinoblastoma (RB), NSUN2 depletion impairs glycolysis by reducing the stability of HKDC1 mRNA, which is dependent on its m⁵C modification. This regulatory mechanism is shared by the m⁵C reader YBX1, indicating a coordinated role in promoting HKDC1 expression^111^. In addition to the above two kinds of RNA methylation modifications that have attracted much attention, the deeper regulatory mechanisms of m¹A and m⁷G modifications in cancer have also been gradually discovered. Chen *et al.* found that elevated METTL1 levels in anlotinib-resistant OSCC cells contributed to enhanced global mRNA translation and stimulated oxidative phosphorylation (OXPHOS) through m⁷G tRNA modification^112^. METTL1 enhances the stability of PFKFB3 mRNA in an m⁷G-dependent manner. Consequently, the up-regulated PFKFB3 augments glycolysis in EC cells by increasing the expression of HK2 and LDHA. This metabolic reprogramming, in turn, contributes to the acquisition of radiotherapy resistance^113^. circFAM126A can bind to HSP90 and promote its ubiquitination to reduce expression, and then inhibit the expression of downstream target AKT1, and finally inhibit glycolysis. This inhibitory effect is due to the fact that TRMT10C increases the stability of circFAM126A in an m⁷G-dependent manner^114^. In melanoma, POU4F1 overexpression induces lactate production and glucose uptake while suppressing the infiltration of anti-tumor immune cells (CD8+ T cells, M1 macrophages, and NK cells), thereby promoting anti-PD-1 resistance. This effect is mechanistically driven by POU4F1-mediated upregulation of METTL1, which increases m⁷G methylation on PKM2 mRNA to enhance glycolysis^115^. METTL1 stabilizes PKM mRNA in a m⁷G-dependent manner, leading to upregulated PKM2 expression. This enhancement drives glycolysis and lactate production. The resulting lactate subsequently induces METTL1 expression, establishing a positive feedback loop that sustains this metabolic circuit^116^. Meanwhile, ALKBH3 promotes the expression of ATP5D to promote the glycolysis process^117^. In Doxorubicin (Dox)-resistant TNBC cells, ALKBH3 elevates m^1^A enrichment on the 3'UTR of ALDOA mRNA. This modification enhances the stability of ALDOA transcripts without affecting their translation efficiency, thereby boosting glycolysis and conferring greater chemoresistance in these drug-resistant cells^118^.

## 4. RNA Methylation Modifications Regulate Lipid Metabolism in Cancer

**Figure 4:**
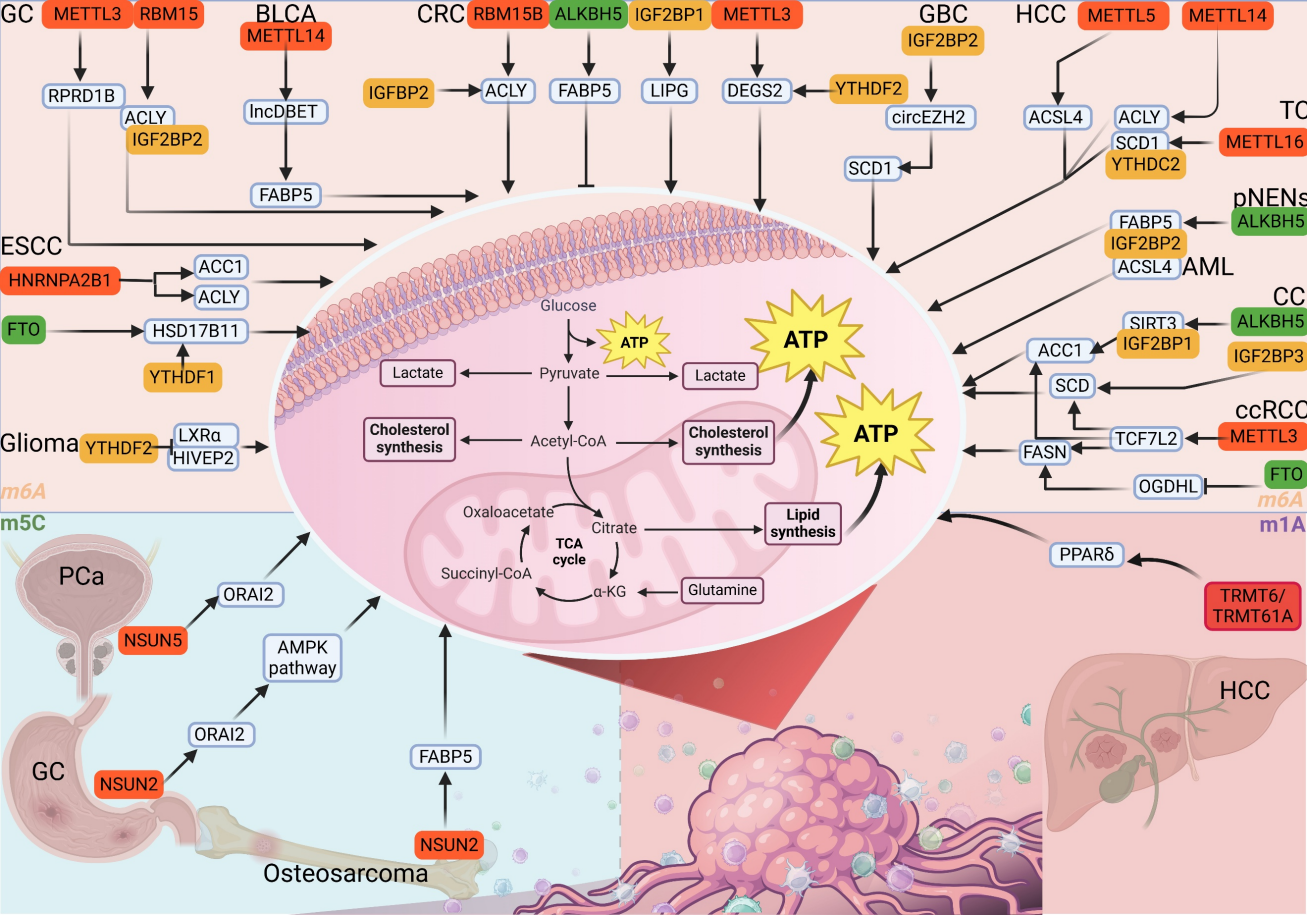
Regulatory mechanisms of RNA methylation in lipid metabolism. (Upper section): m⁶A Modification in Cancer Lipid Metabolism. The m⁶A writers (including METTL3, METTL5, METTL14, RBM15, METTL16 and HNRNPA2B1) and readers (YTHDF1-2 and IGF2BP1-3) upregulate the expression of RNAs involved in lipid metabolism, thereby promoting enhanced lipogenesis. Conversely, the m⁶A eraser FTO and ALKBH5 also upregulate lipid metabolism and facilitates cancer progression. (Lower left quadrant): m⁵C-Mediated Regulation. The m⁵C methyltransferase NSUN2 orchestrates lipid metabolism in Prostate cancer, Gastric cancer (via ORAI2 modulation) and Osteosarcoma (through FABP5 regulation). (Lower right quadrant) m¹A-Dependent metabolic reprogramming in HCC. The writer complex TRMT6/TRMT61A governs lipid metabolic rewiring in hepatocellular carcinoma by modulating the PPARδ signaling axis.

Lipid metabolism plays a key role in human growth and development, and abnormal metabolic metabolism of these substances often leads to a variety of diseases^119^. In this section, we emphasized the indelible role of lipid metabolism mediated by RNA methylation in human cancers (Table 2) (Fig. 4).

m⁶A methylation plays an important role in the regulation of lipid metabolism in tumor cells. For example, HNRNPA2B1 promotes the expression of ACLY and ACC1 and induces the synthesis and deposition of lipids in ESCC^120^. FTO affects the expression of HSD17B11 through YTHDF1-mediated m⁶A modification, and low expression of YTHDF1 can increase the expression of HSD17B11 to inducing the formation of lipid droplets in ESCC^121^. METTL3 can increase the stability of RPRD1B mRNA, which can bind to the promoter of c-Jun and c-Fos to activate their expression, and then promote the expression of SREBP1, a key molecule in the synthesis of fatty acids and triacylglycerol^122^. In GC, RBM15 regulates ACLY mRNA in an IGF2BP2-dependent manner, thereby increasing its expression and enhancing tumor cell adipogenesis^123^. In CRC, it was found that IGFBP2 can increase the stability of GPX4 mRNA to promote its expression, and then activate cyclic GMP-AMP synthase (cGAS-STING) signaling pathway to inhibit lipid peroxidation^124^. ALKBH5 promotes the expression of FABP5 mRNA in CRC via m⁶A modification. Moreover, FABP5 can interact with fatty acid synthase (FASN) and reduce lipid synthesis^125^. In CRC, IGF2BP1 could increase LIPG mRNA stability to promote lipid metabolism^126^. Guo *et al.* reported that knockdown of METTL3 reduced the m⁶A modification of DEGS2 mRNA and upregulated its mRNA levels, which further promoted ceramide synthesis^127^.

METTL3/METTL14 can enhance the expression of ACLY and SCD1 through m⁶A modification, thereby activating lipid synthesis and accumulation in liver cancer cells^128^. In addition, METTL5 is upregulated in liver cancer, and the translation of ACSL4 molecule is also promoted to activates lipid synthesis, resulting in increased contents of triglycerides, free fatty acids and cholesterol^129^. In gallbladder cancer (GBC), IGF2BP2 is able to recognize the m⁶A modification site of circEZH2 and stabilize its expression, and subsequently upregulate SCD1 to promote lipid metabolic reprogramming^130^. ALKBH5 is highly expressed in pancreatic neuroendocrine neoplasms (pNENs) and promotes the expression of FABP5 by reducing the m⁶A modification degree of FABP5 mRNA at 5'UTR. Subsequently, FABP5 causes lipid accumulation in pNENs via the PI3K/AKT/mTOR axis^131^. In bladder cancer, METTL14 can bind to the specific sites of lncRNA DBET, and enhance its stability. lncDBET then activates the expression of FABP5 and PPARγ to promote the synthesis and deposition of lipid^132^. FTO specifically reduces the m⁶A modification of OGDHL mRNA and reduces its expression. Low expression of OGDHL leads to the accumulation of molecules such as triglycerides and saturated fatty acids^133^. Another study on ccRCC indicates that HIF2α can activate the expression of METTL3, thereby increasing the m⁶A modification of TCF7L2 mRNA, and recognized byYTHDC1 to promote lipid metabolism^134^. METTL16 can enhance the m⁶A modification of SCD1 mRNA and enable it to be recognized by YTHDC2, thereby reducing the expression and weakening the abnormal lipid metabolism in thyroid cancer^135^. In cervical cancer, the downregulation of SIRT3 caused by ALKBH5 leads to the inhibition of the deacetylation process of ACC1, thereby causing the inhibition of synthesis and accumulation of free fatty acids^136^. IGF2BP3 specifically binds to the m⁶A modification site of SCD mRNA in CESC, thus promoting the contents of triglyceride, palmitoleic acid and oleic acid^137^. In addition, IGF2BP2 can increase the expression of PRMT6 mRNA to inhibit MFSD2A, thereby promoting the lipid metabolism of leukemia stem cells (LSCs)^138^. Upregulated YTHDF1 can inhibit the effluence of cholesterol in gliomas cells and promote the intake of cholesterol by inhibiting the expression of LXRα and HIVEP2^139^. Lipid metabolism mediated by RNA methylation plays a crucial role in tumors, especially in promoting the process of lipid synthesis and deposition in tumor cells^140^. Knockdown of NSUN2 can reduce the m⁵C methylation modification of FABP5 and inhibit its expression to weaken the metabolic process of fatty acids in osteosarcoma cells^141^. Omental adipocytes provide fatty acids to peritoneal metastatic gastric cancer cells, thereby activating the AMPK signaling pathway to augment transcription factor E2F1 expression, which in turn upregulates NSUN2. NSUN2 induces m⁵C methylation on ORAI2 mRNA, with these modification sites being recognized by YBX1 to facilitate the stabilization^142^. In prostate cancer, it was found that phosphorylated NSUN5 activates m⁵C modification of ACC1 mRNA, promoting lipid synthesis and deposition^143^. NSUN2 promotes SREBP2 expression by increasing its m⁵C modification in an YBX1-dependent manner, and then promotes cholesterol metabolism in HCC cells^144^. NSUN2-mediated m⁵C modification of SOAT2 promotes cholesterol biosynthesis, facilitating metabolic reprogramming in HCC cells^145^. The m¹A methyltransferase complex formed by TRMT6 and TRMT61As is an important complex in the m¹A modification process^146^. In liver cancer, TRMT6/TRMT61A can promote the cholesterol synthesis process by promoting the translation process of PPARδ mRNA through m¹A modification^147^. Miao *et al.* initially discovered that the expression of TRMT61A in CRC tumor-infiltrating CD8+ T cells was inhibited. Mechanically, TRMT61A can increase the translation of ACLY and further increase the biosynthesis of cholesterol in CD8+T cells^148^.

## 5. RNA Methylation Modifications Regulate Amino Acid Metabolism in Cancer

Amino acid metabolism plays an important role in energy production, maintenance of redox homeostasis, proteins and nucleotide synthesis in cancer cells (Table 3). Increased metabolism of glutamine in tumor cells can provide sufficient energy and substrates used for synthesis for tumor cell proliferation^149^.

In GC, FTO removes the methylation site of circFAM192A to protect it from degradation, and then circFAM192A directly binds to SLC7A5 to enhance the stability, which ultimately promotes leucine uptake^150^. In addition, YTHDF1 promotes glutaminase (GLS) protein synthesis by recognizing GLS mRNA, and then promotes the uptake of glutamine in CRC^151^. Han *et al.* found that YTHDF2 could recognize and bind to ATF4 mRNA, thereby reducing its expression. However, inhibition of glutaminolysis could further upregulate FTO to reduce the m⁶A modification of ATF4 mRNA and avoid the recognition of reader and stabilizing its expression^152^. It has also been found that FTO enhances the expression of SCL7A11 and GPX4 mRNA, thereby enabling the transport of extracellular cysteine into intracellular cysteine, and converting cysteine into oxidized glutathione (GSSG)^153^. In lung cancer, METTL3 promotes glutamine metabolism by upregulating SCL7A5 expression^154^. Another study found that SLC7A5 is recognized and upregulated by IGF2BP2, thereby increasing methionine transport^155^. RBM15 was found to upregulate the expression of serine and glycine metabolism-related proteins by recognizing their mRNAs and promote the progression in breast cancer^156^.

In cervical cancer, it was found that METTL3 increased the metabolism of glutamine in cervical cancer cells through m⁶A methylation of SLC38A1 mRNA^157^. In addition, METTL14 protects endometrial cancer cells from ferroptosis by regulating arginine methylation through binding to PRMT3^158^. YTHDF3 promotes the expression of lysyl oxidase LOXL3 by recognizing its methylated sites in melanoma^159^. IGF2BP2 upregulates the expression of key genes in glutamine metabolism (MYC, GPT2, and SLC1A5) in an m⁶A-dependent manner to promote AML progression^160^. METTL16 upregulates the expression of the branched-chain amino acid transaminases BCAT1 and BCAT2 in an m⁶A-dependent manner, which in turn regulates BCAA metabolism^161^. ESCC cells consume exogenous methionine to produce SAM, which in turn provides a substrate for m⁶A modification. Methionine and SAM increase the m⁶A modification in the 3'-UTR of NR4A2 via METTL3, thereby promoting its expression^162^. YTHDF2 overexpression exhibits oncogenic properties in DLBCL by promoting a ceramide metabolic axis. Specifically, YTHDF2 enhances the stability and expression of ACER2 through m⁶A-dependent regulation. This leads to accelerated hydrolysis of ceramide to sphingosine, and its subsequent conversion by Sphingosine kinase (SphK) to S1P, which activates pro-survival ERK and PI3K/AKT signaling, driving tumorigenesis. The malignant phenotype in DLBCL cells is effectively suppressed by the addition of exogenous ceramide *in vitro*^163^.

In addition to m⁶A methylation, m⁵C methylation, as one of the most important RNA methylation modifications, also plays a role in various cancers^32^. In GC, NR_03392 enhances glutamine metabolism by acting as a scaffold for the IGF2BP3/HUR complex to maintain the mRNA stability of GLS. Interestingly, as a result of elevated glutamine metabolism, accumulation of its metabolite α-KG can positively feedback increase the expression of NR_03392 by demethylating its promoter^164^. Aberrant accumulation of α-KG has been shown to act as a cofactor for DNA demethylases (TETs) and histone demethylases (JMJDs) in regulating the expression of cancer-associated genes, which is one way in which amino acid metabolites exert carcinogenic effects^165^. In pancreatic cancer, ALYREF indirectly promotes the transcription of SLC7A5 by up-regulating JunD in an m⁵C-dependent manner to regulate the amino acid metabolism^166^. In AML, NSUN2 stabilizes the mRNA of phosphoglycerate dehydrogenase (PHGDH) and SHMT2—two key enzymes in the serine/glycine biosynthesis pathway—by regulating m⁵C modification, thereby enhancing the expression of PHGDH^167^. In-depth studies to understand the role of RNA methylation modifications in amino acid metabolism will provide new insights for cancer diagnosis and treatment^168^.

## 6. RNA Methylation Modifications Regulate Other Metabolisms in Cancers

In addition to participating in the metabolic regulation of the three major nutrients in the carcinogenic process, RNA methylation modification can also affect cancer progression by regulating other types of metabolism, such as mitochondrial metabolism and iron metabolism (Table 4).

Ferroptosis is a new type of iron-dependent programmed cell death. Under the action of ferric or ester oxygenase, unsaturated fatty acids with high expression on cell membrane undergo lipid peroxidation, which induces cell death^169^. In GBM, knockdown of C5aR1 can reduce the expression of METTL3, thus weakening the m⁶A modification of GPX4 mRNA and triggering the occurrence of ferroptosis^170^. METTL14 can reduce the stability and expression of FTH1 mRNA in an m⁶A dependent manner, indirectly causing the inhibition of downstream PI3K/Akt signaling pathway, thereby relieving the inhibition of ferroptosis^171^. In non-small cell lung cancer cells, METTL14 enhances mRNA stability of GPX4 to inhibit the ferroptosis^172^. In breast cancer, METTL16 can stabilize the expression of GPX4 by increasing its m⁶A methylation, and GPX4 further reduces the levels of intracellular iron, Fe^2+^ and lipid reactive oxygen species (ROS), inhibits the occurrence of ferroptosis^173^. In EC tissues, HNRNPA2B1 binds to the 3'UTR of FOXM1 mRNA and stabilizes its expression. FOXM1 further binds to the LCN2 promoter and positively regulates the expression to inhibit ferroptosis^174^. In bladder cancer, WTAP can increase m⁶A methylation on the 3'UTR of endogenous antioxidant NRF2 RNA, and subsequently YTHDF1 recognizes the m⁶A site on NRF2 mRNA and enhances NRF2 mRNA stability, inhibiting ferroptosis^175^. HIF-1α induces the lncRNA-CBSLR, which scaffolds the formation of a ternary complex with YTHDF2 and CBS mRNA. This complex promotes CBS mRNA decay, leading to reduced ACSL4 methylation. The hypomethylated ACSL4 protein then undergoes polyubiquitination and degradation, ultimately suppressing ferroptosis^176^. As a reader, NKAP can inhibit ferroptosis in GBM.

Specifically, NKAP can recognize the 5ʹ-UTR of SLC7A11 mRNA and bind to it to stabilize its expression and increase the protein level of SLC7A11^177^. Similarly, the regulation of SCL7A11 by FTO was found to affect glutamine metabolism in thyroid cancer cells to protect the cells from ferroptosis^178^. It was also found that inhibition of SCL7A11 and GPX4 induced ferroptosis in CRC cells. FTO protected CRC cells from ferroptosis by upregulating SCL7A11 and GPX4 through demethylation^153^. In addition, FTO can co-regulate the methylation modification of GPX4 with YTHDF2 to promote the ferroptosis of colorectal cells to play an oncogenic role^179^. Curdione, as a drug, was found to promote ferroptosis in cancer cells by up regulating the expression of METTL14 and YTHDF2, which in turn affected the expression levels of SLC7A11, SLC3A2, HOXA13 and GPX4^180^. In hepatoblastoma, METTL3 and YTHDF2 inhibit the occurrence of ferroptosis by recognizing the methylation site of LATS2 to promote its degradation, which in turn inhibits the YAP1/ATF4/PSAT1 axis^181^. It has been found that NSUN2 increases m⁵C modification of SLC7A11 mRNA in endometrial cancer, thereby maintaining its stability, and the subsequent increased m⁵C modification is recognized by YBX1 and further participates in mRNA stabilization. Ultimately, SLC7A11 promotes malignant progression by inhibiting ferroptosis^182^. Moreover, lactylation upregulates the catalytic activity of NSUN2, leading to enhanced m⁵C modification and stability of GCLC mRNA, which promotes GSH synthesis and ultimately protects gastric cancer cells from ferroptosis^183^. In AML, deficiency of NSUN2 leads to downregulation of both FSP1 mRNA and protein, consequently sensitizing AML cells to ferroptosis. This heightened sensitivity to ferroptotic inducers can be effectively rescued by FSP1 overexpression. As anticipated, the NSUN2 inhibitor MY-1B recapitulates this effect and demonstrates significant anti-leukemic activity^184^. In addition, ALKBH3 promotes ATF4 expression by demethylating m^1^A modifications on its mRNA. Consequently, ALKBH3 knockdown diminishes ATF4 levels, resulting in the transcriptional downregulation of key ferroptosis regulators (SLC7A11, GPX4, FTH1) and ultimately suppressing ferroptosis in AML^185^. NSUN2 and ALYREF promote sorafenib resistance in HCC by orchestrating an m⁵C-dependent axis centered on the lncRNA MALAT1. Specifically, they stabilize MALAT1, which enables the cytoplasmic translocation of ELAVL1. This relocalized ELAVL1 then stabilizes SLC7A11 mRNA, thereby suppressing ferroptosis^186^. Moreover, NSUN2-mediated m⁵C modification of RNF115 mRNA is recognized by YBX1, thereby enhancing its translation, the increased RNF115 then suppresses ferroptosis by promoting K27-linked ubiquitination and autophagic degradation of DHODH^187^. In NSCLC, NSUN2 introduces m⁵C modifications within the 5'UTR of NRF2 mRNA, thereby enhancing its stability. Subsequently, the reader protein YBX1 recognizes and binds to these m⁵C sites, further promoting the upregulation of NRF2 mRNA levels. Consequently, this NSUN2/YBX1-NRF2 axis ultimately enhances ferroptosis resistance in NSCLC cells^188^. He *et al.* found that overexpression of METTL1 led to an increase in mature miR-26a-5p, which further targeted FTH1 mRNA. The reduction of FTH1 significantly increases lipid peroxidation and ferroptosis^189^.

Mitochondrial metabolism also plays a critical role in tumor development. In addition to the most basic function of ATP production, mitochondria are able to provide substrates for anabolic metabolism through apoptosis, and mitochondria are able to generate ROS as well as promote RCD signaling for tumor progression^190^. In ccRCC, FTO promotes the expression of PGC-1α mRNA by reducing the m⁶A methylation level, thereby inducing an increase in mitochondrial activity and ROS generation^191^. In CRC, it has been discovered that RALY, as an RNA-binding protein, can facilitate the post-transcriptional processing of specific miRNAs, such as miR-676, miR-483, and miR-877 with METTL3, thereby leading to increased ATP production and decreased ROS levels within CRC cells^192^. Reduced METTL14 decreases m⁶A modification on pri-miR-17, impairing YTHDC2 binding and subsequently increasing pri-miR-17 stability and mature miR-17-5p levels. The elevated miR-17-5p in turn suppresses MFN2, resulting in diminished mitochondrial fusion, increased fission, and enhanced mitophagy, collectively promoting 5-FU resistance in CRC cells^193^. Additionally, the latest research indicates that FTO can promote the degradation of caveolin-1 through demethylation. Depletion of caveolin-1 significantly increases intracellular ATP levels, ATP synthase activity, and cellular OCR in GC cells. However, in FTO-deficient GC cells, caveolin-1 leads to an increased proportion of mitochondrial fragments, thereby suppressing mitochondrial respiration^194^. In OC cells, lncRNA CACNA1G-AS1 upregulates IGF2BP1 expression and enhances FTH1 expression via m⁶A methylation. Following CACNA1G-AS1 knockdown, mitochondrial volume significantly decreases, accompanied by mitochondrial membrane rupture and cristae disappearance. These findings indicate that CACNA1G-AS1 suppresses mitophagy through the IGF2BP1-FTH1 axis^195^. WTAP upregulates ULK1 expression by enhancing its m⁶A modification. IGF2BP3 recognizes these m⁶A sites and stabilizes ULK1 mRNA, which in turn enhances mitophagy, thereby promoting proliferation and migration of OC cells^196^. In SCLC, METTL3 overexpression increases m⁶A modification of DCP2, resulting in downregulation of DCP2 protein. This downregulation in turn impairs the degradation of Pink1 and Parkin, enhances mitophagy, and ultimately confers chemotherapy resistance^197^.

Recent research indicates that NSUN4 upregulates the expression and nuclear export of circERI3 via m⁵C modification, which consequently interacts with DDB1 to facilitate PGC-1α transcription in lung cancer cells. Silencing circERI3 led to elevated ROS levels, a decrease in mitochondrial number and overall mitochondrial dysfunction. These detrimental effects were reversed by PGC-1α, which ultimately boosted mitochondrial energy metabolism^198^. In lung cancer, NSUN2 mediates m⁵C methylation of circRREB1, which is recognized by the reader protein ALYREF to facilitate its nuclear export. The exported circRREB1 subsequently stabilizes HSPA8 protein by inhibiting its ubiquitination. This stabilization in turn upregulates the PINK1/Parkin pathway and enhances mitophagy to promote tumor progression^199^. YTHDF2 drives malignant transformation of pre-B cells and induces aggressive B-ALL *in vivo*. Mechanistically, YTHDF2 recruits PABPC1 to recognize and stabilize F-type ATP synthase subunits in an m⁵C-dependent manner. This process enhances mitochondrial OXPHOS^200^. Mitochondrial morphology is closely associated with bioenergetics. PCIF1 was found to regulate lysophosphatidic acid (LPA) levels within mitochondria via LPP3, thereby inhibiting mitochondrial fission and inducing elongation. This remodeling significantly augments mitochondrial respiration, ultimately driving RCC progression^201^. METTL1 and its cofactor WDR4 form an m⁷G methylation complex that modifies tRNA substrates and enhances tRNA expression. Beyond tRNA regulation, the METTL1/WDR4 complex also promotes mitochondrial OXPHOS in GC cells by increasing SDHAF4 expression in Electron Transport Chain (ETC) Complex II through m⁷G modification^202^. TRMT61A can up-regulate the expressions of p-PI3K, CPT1A and CPT1B in prostate cancer through m¹A modification, promote lipidβ-oxidation in mitochondria to enhance mitochondrial metabolism^203^.

## 7. The Interplay between RNA Methylation and the Tumor Microenvironment

### 7.1 Hypoxia-driven RNA methylations in metabolic reprogramming

The hypoxic tumor microenvironment acts not as a mere barrier but as an active orchestrator of cancer progression, driving profound adaptive shifts in cellular behavior^204^. A cornerstone of this adaptation is metabolic reprogramming, exemplified by the Warburg effect, which sustains energy and biomass production under hypoxia^205^. Under hypoxic conditions in GC, HIF-1α transcriptionally induces lncRNA-CBSLR, which subsequently serves as a molecular scaffold to recruit the m⁶A reader YTHDF2 and facilitate its binding to CBS mRNA, leading to downregulation of CBS protein. The reduction in CBS impairs the methylation of ACSL4, thereby targeting ACSL4 for polyubiquitination and proteasomal degradation. As ACSL4 is a key promoter of ferroptosis, its degradation ultimately suppresses ferroptotic cell death^176^. The hypoxic tumor microenvironment (TME) in ccRCC instigates profound metabolic rewiring via RNA methylation. Central to this pathway is the sustained activation of HIF2α, which transcriptionally upregulates METTL3. Consequently, METTL3-dependent m⁶A methylation stabilizes TCF7L2 mRNA, elevating its protein expression. TCF7L2, in turn, functions as a metabolic switch to activate Wnt signaling and trigger de novo fatty acid synthesis. This hypoxia-driven metabolic shift enhances acetyl-CoA production, which facilitates histone acetylation and EMT, thereby providing pivotal support for tumor cell survival and dissemination under these adverse conditions^134^. Hypoxia triggers a feed-forward circuit in pancreatic cancer where the m⁶A demethylase ALKBH5, through demethylating HDAC4 mRNA, enables HDAC4-mediated stabilization of HIF1α. The activated HIF1α then further elevates ALKBH5 transcription, ultimately establishing a self-reinforcing ALKBH5-HDAC4-HIF1α loop that drives persistent glycolytic reprogramming^206^. In bladder cancer, HIF-1α directly transactivates the m⁶A reader ALYREF under hypoxic conditions. Upregulated ALYREF in turn binds to and stabilizes the mRNA of PKM2, leading to its augmented expression. This HIF-1α/ALYREF/PKM2 axis thereby accelerates glycolytic flux, fulfilling the bioenergetic and biosynthetic demands for bladder tumorigenesis.

### 7.2 RNA methylation in immune cell infiltration and function

Beyond its intrinsic role in hypoxic tumor microenvironment, RNA methylation exerts a profound 'inside-out' effect on the TME by modulating the expression of immunoregulatory molecules. In PDAC, elevated matrix stiffness stabilizes IGF2BP2 by reducing its ubiquitination. Stabilized IGF2BP2 then binds to m⁶A sites on SGMS2 mRNA, promoting its expression and enhancing sphingomyelin synthesis. This increase subsequently facilitates immune escape by promoting the localization of PD-L1 onto membrane lipid rafts, ultimately blunting tumor cell susceptibility to T cell-mediated killing^207^. In various tumor cells, YTHDF2 has been found to regulate immune escape. Mechanistically, the YTHDF2 deficiency significantly increases the m⁶A level in the 3'UTR region of CX3CL1, thereby promoting its expression. This upregulation, in the presence of Interferon-gamma (IFN-γ), facilitates the polarization of anti-tumor macrophages. Additionally, YTHDF2 deficiency suppresses tumor glycolysis, which enhances mitochondrial respiration in CD8^+^T cells and subsequently stimulates their anti-tumor function. As a key effector cytokine produced by CD8^+^T cells, IFN-γ can downregulate YTHDF2 expression in tumor cells, thereby inhibiting tumor development at its source^208^. Specific ablation of USP47 in Treg cells augments anti-tumor immunity and disrupts Treg homeostasis. Mechanistically, USP47 stabilizes the m⁶A reader YTHDF1 via its deubiquitinase activity. Stabilized YTHDF1 then binds to m⁶A-modified c-Myc mRNA and promotes its decay, thereby repressing c-Myc protein synthesis and its-driven glycolytic flux, which ultimately attenuates the anti-tumor T cell immune response^209^. The m⁶A reader YTHDF1 exerts its function by post-transcriptionally stabilizing MCT1 mRNA in an m⁶A-dependent manner, leading to the augmentation of MCT1 expression. The upregulation of MCT1, a key lactate transporter, exacerbates glycolytic metabolism and lactate accumulation within the tumor microenvironment. This metabolic reprogramming creates an immunosuppressive niche by simultaneously dampening the cytotoxic activity of infiltrating CD8^+^T cells and stimulating the surface expression of PD-L1 on tumor cells, thereby enabling the cancer cells to escape immune surveillance^210^. CRC cells, when co-cultured with macrophages, reprogram macrophage metabolism by enhancing fatty acid oxidation (FAO). This metabolic shift is facilitated by the demethylase ALKBH5, which, through the removal of m⁶A modifications, upregulates the expression of CPT1A-a key rate-limiting enzyme in FAO. The ALKBH5-mediated upregulation of CPT1A consequently drives fatty acid metabolic reprogramming and promotes M2 macrophage polarization, ultimately fostering CRC progression^211^. By directly binding to and stabilizing MCT4 mRNA in an m⁶A-dependent manner, hnRNPA2B1 upregulates MCT4 expression. This enhances lactate efflux from tumor cells, thereby acidifying the tumor microenvironment, which subsequently suppresses immune cell cytotoxicity and fosters tumor immune escape^212^. In GBC, IGF2BP2 upregulates PRMT5 by enhancing its m⁶A modification. The increased PRMT5 activates SREBP1, thereby upregulating fatty acid synthases (e.g., ACC1, FASN, SCD1), driving lipid biosynthesis and accumulation. This metabolic alteration subsequently remodels the tumor immune microenvironment, resulting in expanded populations of myeloid-derived suppressor cells (MDSCs) and Tregs and diminished CD8+ T cell infiltration, which together facilitate immune escape^213^.

Within the TME, elevated cholesterol drives CD8^+^T cells to excessively take up fatty acids, which in turn induces lipid peroxidation and ferroptosis. This chain of events severely compromises the cytotoxic function of CD8^+^T cells, thereby promoting immune escape. In HCC, the overexpression of NSUN2 in cancer cells augments this process. NSUN2 mediates m⁵C methylation to stabilize SOAT2 mRNA and enhance its transcription, leading to increased intracellular cholesterol levels that contribute to the suppression of CD8^+^T cell function^145^. In PDAC, the m⁵C reader ALYREF binds to m⁵C-modified JunD transcripts, enhancing their stability. This post-transcriptional regulation leads to an accumulation of JunD protein, which subsequently upregulates the expression of SLC7A5. When overexpressed on the surface of tumor cells, SLC7A5 competitively depletes essential amino acids from the TME. This nutrient deprivation impairs the infiltration and function of CD8+ T cells, thereby facilitating immune evasion^166^. In ccRCC, the loss of NSUN2 abrogates ENO1 mRNA stability via m⁵C modification, thereby suppressing the glycolytic-lactate pathway and subsequent histone H3K18 lactylation. This epigenetic change inhibits the TOM121/MYC/PD-L1 axis, leading to reduced PD-L1 expression and a consequent enhancement of CD8+ T cell-mediated tumor killing^109^.

METTL1 installs m⁷G modifications within the PKM transcript, specifically promoting the expression of the PKM2 isoform. This enhanced PKM2 expression accelerates glycolysis and lactate production, which in turn drives histone lactylation, particularly at the H3K9la. Notably, this epigenetic mark transcriptionally activates METTL1 itself, establishing a potent feedforward loop-the METTL1/PKM2/H3K9la axis-that vigorously sustains glycolytic flux in CRC cells. Beyond metabolic reprogramming, METTL1 also significantly upregulates the transcription of the immune checkpoint molecule CD155. The elevated CD155 expression on tumor cells contributes to immune evasion by suppressing the proportion and function of CD16^+^NK cells^116^.

### 7.3 RNA methylations and metabolic reprogramming under nutrient stress

The m⁶A reader IGF2BP3 plays a pivotal role in sustaining the stemness of AML cells by reprogramming serine metabolism. It directly binds to and stabilizes the mRNA transcripts of key serine synthesis pathway (SSP) genes, including ATF4, PHGDH and PSAT1. This post-transcriptional regulation drives high intracellular serine synthesis. The newly synthesized serine is then funneled into one-carbon metabolism, which generates antioxidants like glutathione. This metabolic reprogramming constitutes the chemical basis for maintaining the leukemic stem cell state. Therapeutically, depleting IGF2BP3 sensitizes AML cells to serine and glycine (SG) deprivation, effectively suppressing tumor progression^214^. Under glucose deprivation, HCC cells exhibit reduced m⁶A methylation in exon 1 and the 5'-UTR regions of FOSL1 mRNA, which attenuates its mRNA decay and consequently upregulates FOSL1 expression. Furthermore, FOSL1 directly suppresses the transcription of ATF3, leading to decreased formation of the ATF3-MAFF heterodimer. Thus, FOSL1-mediated repression of ATF3 enhances the transcriptional activity of NRF2, thereby augmenting the antioxidant response and alleviating glucose deprivation-induced ROS accumulation, which in turn mitigates necrotic cell death in hepatoma cells^215^

## 8. Potential Applications of RNA Methylation in Cancer

### 8.1 Effects of RNA modifications on radiotherapy response

RNA modifications can exert a significant influence on the efficacy of radiation therapy in cancer treatment. Zhao *et al.* used various methodologies, including RNA sequencing and quantitative real-time polymerase chain reaction, and demonstrated that m⁶A-modified eRNA facilitated the resistance of bone-metastatic prostate cancer to radiotherapy^216^. Additionally, Visvanathan *et al.* reported that the GBM cells with silenced METTL3 exhibited increased sensitivity to γ irradiation and decreased DNA repair efficiency^217^. Furthermore, FTO in cervical squamous cell carcinoma can enhance radiotherapy resistance by modulating the expression of β-catenin through the downregulation of m⁶A-modified mRNA transcription^217^. In addition to m⁶A modification, m⁵C modification modulates radiotherapy response. Niu *et al.* suggested that the presence of cis-eQTL in NSUN2 increases resistance to radiotherapy in ESCC through mRNA-m⁵C methylation^218^. And Yu *et al.* found that NSUN6 promoted the radioresistance of cervical cancer cells by regulating the m⁵C modification of NDRG1^219^.

### 8.2 Effects of RNA modifications on chemotherapy response

Diverse cancer cell types use RNA modification to counteract cell death induced by chemotherapeutic drugs. Shi *et al.* reported that YTHDF1 upregulation in non-small cell LC was associated with a positive clinical prognosis. Conversely, YTHDF1 downregulation increased the cisplatin therapy resistance of cancer cells^220^. Similarly, Wang *et al.* found that NSUN2 induced gefitinib resistance through the YBX1/QSOX1 axis in lung cancer cells^221^. Additionally, Gao *et al.* demonstrated that YBX1 underwent SIAH1-mediated ubiquitination at the lysine residue located at position 304, resulting in the enhanced sensitivity of epithelial ovarian cancer cells to cisplatin^222^. In addition, YBX1 was also found to resist cisplatin-induced oxidative stress in ovarian cancer cells by affecting CHD3 expression^223^. The knockdown of METTL3 in PC and YTHDF1 in CRC enhanced the sensitivity of cancer cells to 5-fluorouracil^224^. However, NSUN2 upregulation in prostate cancer cells decreased the responsiveness of cancer cells to 5-fluorouracil treatment^225^. Additionally, ALKBH5 overexpression significantly potentiated the sensitivity of PDAC cells to gemcitabine^226^. In gemcitabine-resistant PDAC cells, METTL14 is significantly upregulated and increases m⁶A modification at the 3'UTR of TGFB2 mRNA, which is recognized by IGF2BP2 and stabilizes its expression. High expression of TGFB2 increases gemcitabine-resistant PDAC cells' drug resistance^227^. In breast cancer, Liu *et al.* found that AK4 regulated by m⁶A could increase the resistance of breast cancer cells to tamoxifen by increasing intracellular ROS and inhibiting mitochondrial apoptosis^228^. METTL3 mediates mRNA shear of precursor ESRRG through m⁶A modification, which leads to high expression of ERRγ, and at the same time, ERRγ can bind to CPT1B promoter to activate its transcription and induce chemical resistance of tumor mediated by mitochondrial FAO process in breast and liver cancer cells^229^.

### 8.3 Effects of RNA modifications on immunotherapy response

RNA methylation is increasingly recognized as a pivotal modulator of anti-tumor immunity-the immune system's ability to target and remove tumor cells. It orchestrates this effect by regulating immune checkpoint proteins and reshaping the TME, thereby shaping immunotherapy efficacy^230^. Given this role, RNA methylation markers are being harnessed to predict responses to immunotherapy. In HCC, the m⁶A reader HNRNPA2B1 was significantly upregulated in patients responsive to immunotherapy (P=0.0062). Although high HNRNPA2B1 expression is generally associated with poorer prognosis in HCC, it paradoxically correlated with longer overall survival in the IMvigor210 cohort receiving anti-PD-L1 therapy (P=0.047)^231^. In HNSCC, an RNA Modification Score (RMscore) was developed from the expression of 26 readers and effectively dichotomized patients into two modification patterns. A low RMscore proved to be a powerful predictor of favorable immunotherapy outcomes, conferring a survival advantage (median OS: 9.89 vs 5.13 months) and a superior objective response rate (26% vs 5%)^232^. A study by Wang *et al.* established a multi-RNA methylation (m⁶A/m⁵C/m¹A) prognostic signature in cervical cancer. Their analysis revealed that the high-risk group had a greater probability of responding to anti-CTLA-4 therapy, along with reduced IC50 values, indicating these patients represent a potential target population for this treatment strategy^233^. Stratification of soft tissue sarcomas by m⁶A-related metabolic pathways reveals two molecular subtypes. Subtype A is characterized by enhanced anti-tumor immunity and greater susceptibility to immunotherapy and chemotherapy^234^. Using key m⁵C RNA methylation regulators (DNMT1, NSUN4, and NSUN7), Zhang *et al.* developed a prognostic signature for rectal adenocarcinoma (READ), which stratified patients into high- and low-risk groups. The low-risk group demonstrated higher expression of immune checkpoint molecules and a greater proportion of complete/partial responses, indicating heightened sensitivity to immune checkpoint inhibitor therapy^235^.

Beyond its role in predicting therapeutic response, RNA methylation also plays a pivotal role in modulating tumor-derived immunosuppressive factors, thereby governing the mechanisms of tumor immune evasion^236^. In bladder cancer cells, inhibition of the JNK signaling pathway downregulates METTL3 expression. This METTL3 deficiency, in turn, reduces the m⁶A modification and expression of PD-L1 by diminishing the stability of its mRNA. Consequently, this cascade enhances the anti-tumor immune response *in vivo*^237^. Evidence suggests that SHMT2 knockdown inhibits EC tumorigenesis. SHMT2 augments the m⁶A modification of c-Myc via METTL3, thereby facilitating its recognition and stabilization by IGF2BP2. This pathway sustains c-Myc overexpression, which consequently upregulates PD-L1 to promote immune evasion in EC cells^238^. ALKBH5 loss fosters a more immunostimulatory microenvironment in glioma through an expanded T-cell presence-marked by increased CD4^+^and CD8^+^T lymphocyte counts and a higher CD8^+^/CD4^+^ ratio-coupled with a reduction in PD-L1 protein expression^239^. In PCa cells, overexpression of YTHDF1 drives tumor immune evasion by upregulating the expression of PD-L1 on the cell surface^240^. It has been shown that in breast cancer, METTL3 catalyzes m⁶A modification on PD-L1 mRNA. This m⁶A mark is then recognized by IGF2BP3, which binds to and stabilizes the transcript, ultimately leading to augmented PD-L1 expression^241^. In parallel, FTO upregulates PD-L1 under hypoxic conditions through activation of the PDK1/AKT/STAT3 signaling axis^242^. It has been demonstrated that in NSCLC, ALKBH5 downregulates the m⁶A modification of JAK2, resulting in its elevated expression. The subsequent activation of the JAK2/p-STAT3 pathway ultimately drives the induction of PD-L1^243^. It has been shown that YTHDF1 overexpression promotes EHD1 expression by stabilizing its mRNA through an m⁶A-dependent mechanism. The elevated EHD1 binds to PD-L1, inhibits its lysosomal degradation, and consequently leads to the accumulation of PD-L1 on the lung adenocarcinoma (LUAD) cell surface, which facilitates immune escape and resistance to ICB treatment^244^. NSUN2 and ALYREF facilitate immune evasion in NSCLC by increasing PD-L1 mRNA expression via m⁵C modification, thereby impairing CD8^+^ T cell infiltration in the TME. The finding that NSUN2 depletion sensitizes tumors to immunotherapy highlights this pathway's therapeutic relevance^245^. METTL3 enhances the m⁶A modification of circSLCO1B3, facilitating its recognition by YTHDC1 and subsequent stabilization. This stable circSLCO1B3 protein promotes PD-L1 accumulation and immune evasion in ICC by suppressing the ubiquitin-proteasome pathway, ultimately leading to increased PD-L1 protein expression^246^. YBX1 promotes PD-L1 expression by recognizing m⁵C methylation on STAT1 mRNA and enhancing its stability in ICC cells. This regulation underscores an indirect yet potent pathway of immune checkpoint control^247^. m⁶A modification negatively regulates interferon (IFN) response by modulating the turnover of IFN mRNA^248^. Rubio *et al.* proposed that METTL14 depletion upregulated IFNβ1 production, whereas ALKBH5 depletion downregulated IFNβ1 production^249^. FTO silencing increased the sensitivity of melanoma cells to IFN-γ and enhanced the sensitivity of melanoma to anti-PD1 therapy in murine models^250^. Furthermore, NSUN2 is a potential precise biomarker for immune-checkpoint blockade response with potential applications in targeted therapy for head and neck squamous cell carcinoma^251^. Tao *et al.* reported that the YBX1/PD-L1 axis is a promising therapeutic target for potentiating anti-tumor immunity in HCC^252^.

### 8.4 Drugs targeting RNA modification

An imbalanced m⁶A state can disrupt the normal expressions of oncogenes and tumor suppressors, which facilitates tumorigenesis, metastasis, and drug resistance. Targeting these regulatory proteins with small-molecule inhibitors has therefore surfaced as a promising novel approach in oncology (Table 5). The primary mechanism of these compounds is to reverse the cancer-specific m⁶A imbalance, ultimately suppressing tumor growth and overcoming therapeutic resistance.

From an adenine-based compound library, the potent METTL3 inhibitor UZH1a was identified with an IC50 of 280 nM, reducing global m⁶A levels in mRNA by approximately 70%^253^. In parallel, UZH2, another specific METTL3 inhibitor, achieved a more pronounced reduction of 80% in m⁶A abundance. Notably, neither compound affected other methylation modifications, demonstrating high selectivity^254^. STM2457, a highly specific METTL3/METTL14 inhibitor (IC₅₀=16.9 nM), effectively suppressed global m⁶A modification without inhibiting other methyltransferases. In AML cell lines, it reduced the translation efficiency of oncogenes (SP1, MYC, HOXA10) and inhibited proliferation (IC₅₀ ≈ 1-10 μM). Consistent with these findings, STM2457 (50 mg kg⁻¹) impeded AML growth and extended survival in a PDX mouse model^255^. STC-15, an optimized derivative of STM2457, offers superior potency and metabolic stability. Its inhibition of METTL3 triggers the upregulation of innate immune genes, spurring an anti-tumor response that curbs cancer cell proliferation. This compound has entered early-stage clinical trials (NCT05584111, NCT05605188), representing a milestone as the first-in-class METTL3 inhibitor to reach clinical evaluation, thereby pioneering the therapeutic targeting of RNA modifiers^256^.

The pioneering discovery of FTO inhibitors was reported in 2012 by Chen *et al.*, who identified the natural compound rhein as a first-in-class, competitive inhibitor that targets the catalytic site of FTO in biochemical assays^257^. In leukemia cells, the elevated expression of FTO reduces global m⁶A levels, thereby conferring a resistant phenotype during tyrosine kinase inhibitor (TKI) treatment. Application of rhein to these TKI-resistant cells reverses this effect by increasing m⁶A abundance, subsequently resensitizing them to TKI therapy^258^. Another FTO inhibitor, MO-I-500, functions as a structural mimic of ascorbate—a cofactor that promotes reactions catalyzed by the Fe (II)- and 2-Oxoglutarate (2OG)-dependent dioxygenase family. MO-I-500 exhibits an IC₅₀ of 8.7 µM against purified FTO *in vitro*, and this inhibitory activity corresponded to the suppression of TNBC cell proliferation^259^. A key challenge with first-generation FTO inhibitors like rhein and MO-I-500 is their promiscuous inhibition across the 2OG oxygenase family. A marked improvement in specificity was achieved with compound 12, a 2OG analog that shows 30-fold selectivity for FTO over related enzymes^260^.

Huang *et al.* developed two promising FTO inhibitors, FB23 and its optimized analog FB23-2, which directly bind to FTO and selectively inhibit its m⁶A demethylase activity. Demonstrating the therapeutic potential of targeting FTO, FB23-2 significantly suppressed cell proliferation and, more importantly, induced differentiation and apoptosis in AML cells^261^. Compared to previously reported FTO inhibitors FB23-2 and MO-I-500, the novel compounds CS1 and CS2 demonstrated superior efficacy in suppressing AML cell viability, with IC₅₀ values reduced by 10- to 30-fold. Notably, both CS1 and CS2 significantly inhibited the viability of primary human AML cells while largely sparing healthy counterpart cells, indicating a favorable therapeutic window. Furthermore, FTO inhibition by CS1 or CS2 induced significant apoptosis and cell cycle arrest at the G0 phase in human AML cells^262^. The oncometabolite R-2HG, which is highly produced by mutant isocitrate dehydrogenases 1 and 2 (IDH1/2), exerts its broad intrinsic anti-tumor activity in leukemia and glioma by inhibiting the FTO demethylase. This inhibition leads to an accumulation of global m⁶A modification, which in turn suppresses pro-tumorigenic pathways associated with MYC/CEBPA^263^. In 2015, the non-steroidal anti-inflammatory drug meclofenamic acid (MA) was identified as a novel FTO binder. It acts as a selective FTO inhibitor by competitively occupying the m⁶A substrate-binding site, exhibiting greater selectivity for FTO over its homolog ALKBH5^264^. Subsequently, the ethyl ester derivative of MA (MA2) was developed, which effectively suppressed the growth of glioblastoma stem cells *in vitro* and *in vivo*. Notably, MA2 demonstrated a synergistic therapeutic effect with temozolomide, highlighting a promising combination strategy for glioma treatment^265^.

In parallel with the pursuit of FTO inhibitors, considerable efforts are being devoted to developing small-molecule inhibitors with high selectivity for ALKBH5. MV1035, initially characterized as a sodium channel blocker, was serendipitously identified as a potent off-target inhibitor of ALKBH5 through unbiased screening. Functional validation confirmed that direct incubation of MV1035 with ALKBH5 *in vitro* elevated global m⁶A levels. Consequently, this off-target activity translated into significant inhibition of migration and invasion in glioblastoma models^266^. Based on the X-ray crystal structure of ALKBH5, a computer-based screening approach led to the discovery of ALK-04, a specific inhibitor of this demethylase. Notably, ALK-04 potently sensitizes both colon cancer and melanoma cell lines to anti-PD-1 treatment^267^. Expanding the arsenal of ALKBH5 inhibitors, a separate high-throughput virtual screening of 144,000 compounds identified two effective candidates: 2-[(1-hydroxy-2-oxo-2-phenylethyl) sulfanyl] acetic acid (3) and 4-[268]-1,2-diazinane-3,6-dione (6). Functionally distinct from the immunomodulatory role of ALK-04, these inhibitors were shown to exert antiproliferative effects on three distinct leukemia cell lines at low micromolar concentrations, underscoring their potential in direct anti-cancer applications^268^.

The YTHDF proteins recognize m⁶A through a conserved hydrophobic pocket that accommodates the methylated base embedded within the RRACH consensus sequence. The organic selenium compound, ebselen, has been identified as the first inhibitor that targets this YTH domain. It functions by directly binding to the m⁶A recognition pocket, thereby competitively disrupting the interaction between YTHDF proteins and their target mRNAs within cells^269^. The YTHDF2 inhibitor DC-Y13-27 enhances the anti-tumor effects of radiotherapy and radio-immunotherapy by mimicking the phenotypic consequences of YTHDF2 loss, which include altered MDSC differentiation, suppressed intra-tumoral trafficking, and attenuated immunosuppressive activity^270^. By interacting with a hydrophobic surface at the boundary of the IGF2BP1 KH3 and KH4 domains, Compound 7773 inhibits Kras RNA binding. Consequently, it reduces the levels of Kras mRNA and protein, thereby suppressing downstream signaling, migration, and transformation in cell-based assays without inducing toxicity^271^.

### 8.5 RNA methylation biomarkers for diagnosis and prognosis

Beyond the analysis of tumor tissues, the emerging role of RNA methylation biomarkers in liquid biopsy-particularly from peripheral blood-holds immense promise for revolutionizing cancer prognosis and therapy monitoring. Unlike traditional invasive biopsies, which pose practical challenges for repeated assessment, the detection of circulating RNA methylation signatures offers a minimally invasive, dynamic, and real-time window into the tumor's molecular landscape. This approach allows for the continuous monitoring of disease progression, treatment response, and the early detection of resistance, which is crucial for adapting therapeutic strategies.

In patients with GC, the level of m⁶A in peripheral blood RNA was significantly elevated compared to healthy controls, with an area under the curve (AUC) value of 0.929, substantially outperforming conventional biomarkers such as CEA and CA19-9. Among m⁶A-related enzymes, the expression of two erasers, ALKBH5 and FTO, was markedly downregulated and exhibited a significant inverse correlation with distant metastasis and advanced tumor stage. When m⁶A was combined with ALKBH5 and FTO, the diagnostic performance of the biomarker panel was further improved, achieving an AUC of 0.946^272^. Likewise, a significant increase in m⁶A levels within peripheral blood immune cells was observed in CRC patients, which excellently discriminated CRC from healthy individuals with an AUC of 0.946. Notably, IGF2BP2 alone also showed considerable diagnostic value, achieving an AUC of 0.795, thereby performing on par with conventional serum tumor markers^273^. Furthermore, in addition to m⁶A, CRC patients exhibit a marked increase in m⁵C levels within peripheral immune cells, which correlates positively with disease progression and demonstrates superior diagnostic power (AUC=0.888) over current standard biomarkers like CEA, CA125, and CA19-9. Mechanistically, this elevation in m⁵C was attributed to the roles of NSUN5 and YBX1^274^. Leukocyte m⁶A levels serve as a potent biomarker in NSCLC, showing significant elevation that correlates with disease progression and declines post-resection. It effectively discriminates LUAD (AUC=0.736) and, more strikingly, LUSC with an AUC of 0.963, alongside perfect sensitivity (100%) and high specificity (85.7%), outperforming traditional markers. The underlying mechanism involves a synergistic effect of methyltransferase complex upregulation and demethylase (FTO/ALKBH5) downregulation^275^. In contrast, the level of m⁵C modification in leukocytes was significantly reduced in NSCLC patients, showing a progressive decrease with advancing tumor stage. Diagnostically, m⁵C achieved an AUC of 0.912, outperforming conventional serum tumor markers. Furthermore, a panel combining m⁵C with these established markers enhanced the diagnostic performance, elevating the AUC to 0.960^276^. The m⁶A modification in peripheral blood RNA served as a robust diagnostic biomarker for BC, showing significant elevation in patients versus controls and a positive correlation with advanced stage. It achieved an AUC of 0.887, outperforming CEA and CA15-3. Mechanistically, the upregulation of METTL14 and concurrent downregulation of FTO were identified as potential drivers of the global m⁶A hypermethylation. Notably, a diagnostic model integrating m⁶A with its regulators METTL14 and FTO achieved an even higher AUC of 0.929 and a specificity of 97.4%^277^.

## 9. The Complex Roles of RNA Methylation in Cancer Metabolism

With the deepening investigation of RNA methylation in cancer, a growing body of evidence indicates that RNA methylation exhibits a dual nature. Specifically, the same regulatory machinery-writers, erasers, and readers-can exert either potent oncogenic functions or act as critical tumor suppressors in different cellular contexts^278^. For instance, the core writer METTL3 frequently acts as a clear oncogene in malignancies such as GC and CRC^53^, ^59^. In contrast, Nitin Raj *et al.* identified a role for METTL3 in potentiating the tumor-suppressive activity of p53, as evidenced in *in vivo* mouse cancer models and human cancer cells^279^. Similarly, the eraser ALKBH5 also demonstrates this dual role, it can promote tumorigenesis by maintaining cancer stem cell populations and fostering therapy resistance in certain cancers^185^, while in other contexts, its activity appears to sensitize cells to chemotherapeutic agents or modulate the immune microenvironment, highlighting its remarkably context-dependent functionality^280^. The mechanisms underlying these conflicting findings are multifaceted. First, target gene specificity plays a decisive role, the functional consequences of RNA methylation are entirely dependent on the type of target transcript. In one tumor type, methylation may predominantly destabilize a set of tumor-suppressive mRNAs, leading to an oncogenic outcome^278^. Second, the cellular and molecular context is critical, the overall effect of RNA methylation interacts with cell type-specific signaling networks, genetic mutations, and the tumor microenvironment. Factors such as hypoxia, oncogenic stress, and the status of other epigenetic regulators can reprogram the epitranscriptome, thereby altering the functional output of RNA methylation modifiers^281^. Thus, advancing future therapies requires a shift from simplistic biomarker expression to a mechanism-informed classification of the functional epitranscriptomic state within each tumor.

Although numerous studies have established a close link between RNA methylation and cancer metabolic reprogramming, the central controversy of whether these modifications function as active drivers or secondary consequences of oncogenic signaling remains to be reconciled^282^. Evidence supporting their driver role demonstrates that specific methylation events can directly regulate metabolic flux. For instance, NSUN2 enhances the m⁵C modification on the PGK1 mRNA, which is recognized by YBX1, leading to the upregulation of PGK1 and thereby directly promoting glycolysis^103^. On the other hand, a compelling perspective posits that RNA methylation often serves as a secondary outcome of oncogenic signals, with its global patterns being reshaped by master oncogenes such as MYC and subjected to feedback regulation by metabolic stress^283^. In summary, RNA methylation is more likely a dynamically integrated component within the oncogenic signaling network-it can be activated by upstream signals and, in turn, reinforce tumor malignant phenotypes through downstream metabolic regulation, thereby establishing a positive feedback loop.

## 10. Conclusion and Perspectives

The pivotal role of RNA methylation in reprogramming cancer metabolism has firmly established it as a central layer of epigenetic regulation in oncology. These modifications, mediated by a complex network of 'writers, readers, and erasers', exert profound influence over tumorigenesis and cancer progression by dynamically fine-tuning metabolic pathways. The significance of this metabolism-centered RNA epitranscriptomics extends beyond basic biology, holding immense promise for developing novel prognostic biomarkers and targeted therapeutic strategies.

However, current research is constrained by technical hurdles in precisely mapping various RNA modifications and quantifying their stoichiometry at a single-cell resolution within the complex TME. Furthermore, our understanding of the intricate crosstalk between different RNA modifications and with other epigenetic regulators remains fragmented. A significant shortcoming is the frequent oversight of cell-type-specific functions, particularly the role of RNA methylation in stromal and immune cells within the tumor niche and how it shapes intercellular metabolic communication. Most importantly, the translational gap is substantial, the journey from mechanistic discovery to clinically viable therapeutics targeting the RNA methylome is still in its infancy, hampered by the lack of specific and potent inhibitors.

To address these shortcomings, future researches should be directed along several critical paths. First, the development of novel chemical and sequencing technologies is imperative to achieve spatiotemporal, quantitative, and single-cell resolution mapping of the epitranscriptome. Second, a more systematic investigation is needed to decipher the functional networks of RNA modifications, especially their interplay in the tumor microenvironment that dictates metabolic plasticity and immune evasion. Finally, and most critically, a major frontier lies in accelerating translational efforts. This includes the rigorous validation of RNA modification regulators as druggable targets, the development of selective small-molecule inhibitors or RNA-based therapeutics, and the exploration of their potential in combination with existing modalities like immunotherapy or chemotherapy. In summary, while challenges exist, the rapidly evolving field of RNA methylation and cancer metabolism is poised for groundbreaking discoveries. A concerted effort integrating advanced technologies, sophisticated model systems, and translational research will be essential to fully unravel the mechanistic complexities and, ultimately, harness this knowledge to improve clinical outcomes for cancer patients.

## Figures and Tables

**Table 1:** The role of RNA methylation in glucose metabolism reprogramming in cancer.

RNA methylation type	Cancer type	Methylase	Methylation target	Downstream effectors	Role of RNA methylation target	Cell phenotype	Reference
m⁶A	ESCC	METTL3↑/YTHDF2↑	APC	β-catenin/PKM2	Anti-oncogene	Proliferation	51
m⁶A	ESCC	WTAP↑	PDIA3P1	GLUT1 and HK2	Oncogene	Proliferation, migration, invasion and EMT	52
m⁶A	GC	METTL3↑	HDGF	GLUT4 and ENO2	Oncogene	Proliferation, metastasis and angiogenesis	53
m⁶A	GC	METTL3↑/IGF2BP1↑	NDUFA4	ENO1 and LDHA	Oncogene	Proliferation and apoptosis	54
m⁶A	GC	METTL14↓	LHPP	HIF1A, GLUT1, C-MYC, PDHK1, PKM2, ALDOL A, ENO1, LDHA, and GLS	Anti-oncogene	Proliferation and metastasis	55
m⁶A	GC	WTAP↑	HK2	/	Oncogene	Proliferation, migration	56
m⁶A	GC	KIAA1429↑	LINC00958	GLUT1	Oncogene	Proliferation	57
m⁶A	GC	IGF2BP3↑	c-MYC	/	Oncogene	Proliferation, migration	58
m⁶A	CRC	METTL3↑	HK2 and SLC2A1	/	Oncogene	Proliferation	59
m⁶A	CRC	METTL3↑	GLUT1	mTORC1	Oncogene	Proliferation, clonal formation, G1-phase cell cycle arrest and apoptosis	60
m⁶A	CRC	METTL3↑/YTHDF1↑	HIF-1α	LDHA	Oncogene	Cell viability	61
m⁶A	CRC	METTL3↑	PTTG3P	YAP1	Oncogene	Proliferation	62
m⁶A	p53-WT CRC	METTL14↓/YTHDF2↑	miR-6769b-3p and miR-499a-3p	SLC2A3 and PGAM2	Anti-oncogene	Proliferation	63
m⁶A	CRC	KIAA1429↑	HK2	/	Oncogene	/	64
m⁶A	CRC	YTHDF2↑	circ-0003215	DLG4	Anti-oncogene	Proliferation and metastasis	65
m⁶A	CRC	YTHDF1↑/WTAP↑	FOXP3	SMARCE1	Oncogene	Proliferation, metastasis, migration and invasion	66
m⁶A	HCC	METTL3↑	PDK4	/	Oncogene	Proliferation	67
m⁶A	HCC	METTL14↓	USP48	SIRT6	Anti-oncogene	Cell viability, clone formation, and invasion and migration	68
m⁶A	HCC	ZC3H13↑	PKM2	/	Oncogene	Proliferation, apoptosis, migration and invasion	69
m⁶A	HCC	FTO↑	PKM2	/	Oncogene	Proliferation, colony formation, and G1/G2 arrest	70
m⁶A	HCC	ALKBH5↓	UBR7	Keap1/Nrf2/Bach1/HK2	Anti-oncogene	Proliferation, metastasis, migration and invasion	71
m⁶A	HCC	YTHDF3↑	PFKL	/	Oncogene	Proliferation, migration, invasion, and lung metastasis	72
m⁶A	HCC	IGF2BP2↑	HK2/SLC2A1	/	Oncogene	Proliferation, clone formation	73
m⁶A	CCA	METTL3↑	AKR1B10	/	Oncogene	Proliferation, migration, and invasion	74
m⁶A	PAAD	METTL3↑	HK2	CaMKII/ERK-MAPK pathway	Oncogene	proliferation, neurometastasis, migration, and invasion	75
m⁶A	PAAD	YTHDF3↑	DICER1-AS1	DICER1/miR-5586-5p/SLC2A1, LDHA, HK2, and PGK1	Anti-oncogene	Proliferation and metastasis	76
m⁶A	LCA	YTHDF2↑	6PGD	/	Oncogene	Proliferation	77
m⁶A	LCA	YTHDF1↑	ENO1	/	Oncogene	Proliferation	78
m⁶A	LCA	YTHDF1↑	c-Myc	/	Oncogene	Proliferation and metastasis	79
m⁶A	LCA	METTL3↑/YTHDF1↑	DLGAP1-AS2	c-Myc	Oncogene	Proliferation	80
m⁶A	OSCC	IGF2BP3↑	GLUT1	/	Oncogene	Proliferation, migration and invasion	81
m⁶A	OSCC	IGF2BP2↑	HK2	/	Oncogene	Proliferation, glycolysis, migration and invasion	82
m⁶A	BRCA	METTL3↑	LATS1	Hippo pathway	Anti-oncogene	Proliferation, apoptosis, migration and invasion	83
m⁶A	BRCA	WTAP↑	ENO1	/	Oncogene	Proliferation	84
m⁶A	BRCA	ALKBH5↑	GLUT4	/	Oncogene	Proliferation, migration and invasion	85
m⁶A	CESC	IGF2BP2↑	MYC	/	Oncogene	Proliferation, migration and invasion	86
m⁶A	CESC	FTO↓	HK2	/	Oncogene	/	87
m⁶A	OV	WTAP↑	miR-200	HK2	Oncogene	Proliferation, migration and invasion	88
m⁶A	THCA	ALKBH5↓	circNRIP1	PKM2	Oncogene	Proliferation	89
m⁶A	THCA	FTO↓/IGF2BP2↑	APOE	IL-6/JAK2/STAT3	Oncogene	Proliferation	90
m⁶A	GBM	ALKBH5↑	G6PD	/	Oncogene	Proliferation	91
m⁶A	GBM	FTO↑	PDK1	/	Oncogene	Proliferation, TMZ resistance, apoptosis, DNA damage repair, and glycolysis	92
m⁶A	AML	FTO↑/YTHDF2↑	PFKP and LDHB	/	Oncogene	Proliferation, apoptosis and G0/G1 arrest	93
m⁶A	OS	RBM15↑	HK2, GPI, PGK1	/	Oncogene	Proliferation and invasion	94
m⁶A	ccRCC	IGF2BP1↑	LDHA	/	Oncogene	/	95
m⁶A	RCC	METTL14↓	BPTF	ENO2 and SRC	Oncogene	Metastasis, invasion, migration and EMT	96
m⁶A	DLBCL	WTAP↑ and IGF2BP2↑	HK2	/	Oncogene	Proliferation and induces cell cycle arrest	97
m^5^C	TNBC	NSUN2↑	tRNA^Val-CAC^	/	Oncogene	Proliferation and clonal formation	98
m⁵C	Bca	YBX1↑	TM4SF1	β-catenin/c-Myc signaling pathway	Oncogene	Proliferation, migration, and invasion	99
m⁵C	BCa	ALYREF↑	PKM2	HIF-1α/ALYREF/PKM2	Oncogene	Proliferation	100
m⁵C	LCA	YBX1↑	PFKFB4	/	Oncogene	Proliferation, invasion and migration	101
m⁵C	LCA	NSUN2↑	ME1, GLUT3 and CDK2	/	Oncogene	Proliferation, invasion, migration and angiogenesis	102
m⁵C	GC	NSUN2↑	PGK1	PI3K/AKT pathway	Oncogene	Cell growth, invasion and stemness	103
m⁵C	CRC	NSUN2↑/YBX1↑	ENO1	/	Oncogene	Proliferation, invasion and metastasis	104
m⁵C	HCC	NOP2↑	c-Myc	ENO1, LDHA, PKM2 and TPI1	Oncogene	Proliferation, migration, invasion and metastasis	105
m⁵C	HCC	NSUN2↑	PKM2	/	Oncogene	proliferation and migration	106
m⁵C	ICC	ALYREF↑	PKM2	/	Oncogene	Proliferation, invasion and apoptosis	107
m⁵C	BRCA, PCa, SKCM and CRC	NSUN2↑	TREX2	cGAS/STING	Oncogene	Proliferation, cancer stemness and apoptosis	108
m⁵C	RCC	NSUN2↑	ENO1	TOM121/MYC/PD-L1	Oncogene	Proliferation, migration, invasion and CD8 + T cell infiltration	109
m⁵C	OC	ALYREF↑	BIRC5	/	Oncogene	Proliferation, cell cycle arrest and apoptosis	110
m⁵C	RB	NSUN2↑ and YBX1↑	HKDC1	/	Oncogene	Proliferation and migration	111
m⁷G	OSCC	METTL1↑	tRNA	/	Oncogene	/	112
m⁷G	EC	METTL1↑	PFKFB3	HK2 and LDHA	Oncogene	Proliferation, clonal formation, migration and invasion	113
m⁷G	LCA	TRMT10C↓	circFAM126A	HSP90/AKT1	Anti-oncogene	Proliferation, migration, and apoptosis	114
m⁷G	Melanoma	METTL1↑	PKM2	/	Oncogene	Chemoresistance	115
m⁷G	CRC	METTL1↑	PKM2	CD155	Oncogene	Proliferation and immunosuppression	116
m¹A	CESC	ALKBH3↑	ATP5D	/	Oncogene	Proliferation, angiogenesis, and migration	117
m¹A	TNBC	ALKBH3↑	ALDOA	/	Oncogene	Chemoresistance	118

**Table 2:** The role of RNA methylation in lipid metabolism reprogramming in cancer.

RNA methylation type	Cancer type	Methylase and expression in cancer	Methylation target	Downstream effectors	Role of RNA methylation target	Phenotype	References
m⁶A	ESCC	HNRNPA2B1↑	ACLY and ACC1	/	Oncogene	Proliferation, migration and invasion, tumor size and lymphatic metastasis	120
m⁶A	ESCC	FTO↑/YTHDF1↓	HSD17B11	/	Oncogene	Proliferation, migration and invasion, tumor size	121
m⁶A	GC	METTL3↑	RPRD1B	c-Jun/c-Fos/SREBP1	Oncogene	Migration, invasion and lymphatic metastasis	122
m⁶A	GC	RBM15↑/IGF2BP2↑	ACLY	/	Oncogene	Proliferation, migration and invasion	123
m⁶A/m⁵C	CRC	RBM15B↑, IGFBP2↑ and NSUN5↑	GPX4	cGAS-STING signaling pathway	Oncogene	/	124
m⁶A	CRC	ALKBH5↓	FABP5	FASN	Anti-oncogene	Proliferation, migration and invasion	125
m⁶A	CRC	IGF2BP1↓	LIPG	/	Oncogene	Proliferation, migration and invasion	126
m⁶A	CRC	METTL3↑/YTHDF2↓	DEGS2	/	Oncogene	Proliferation and migration	127
m⁶A	HCC	METTL14↑	ACLY and SCD1	/	Oncogene	Apoptosis and compensatory proliferation	128
m⁶A	HCC	METTL5↑	ACSL4	/	Oncogene	Migration and invasion, tumor size	129
m⁶A	GBC	IGF2BP2↑	circEZH2	miR-556-5p/SCD1	Oncogene	Proliferation, G1/S cell cycle arrest, and tumor growth	130
m⁶A	Pancreatic neuroendocrine neoplasm	ALKBH5↑/IGF2BP2↑	FABP5	PI3K/Akt/mTOR	Oncogene	Proliferation, migration and invasion	131
m⁶A	Bladder cancer	METTL14↑	lncDBET	FABP5/PPARγ	Oncogene	Proliferation, migration and invasion, tumor size	132
m⁶A	ccRCC	FTO↑	OGDHL	FASN	Anti-oncogene	Proliferation, migration, invasion, apoptosis and cell cycle	133
m⁶A	ccRcc	METTL3↑	TCF7L2	FASN, ACC1 and SCD	Oncogene	EMT and metastasis	134
m⁶A	THCA	METTL16↓/YTHDC2↑	SCD1	/	Anti-oncogene	Proliferation and metastasis	135
m⁶A	CSCC	ALKBH5↓	SIRT3	ACC1	Oncogene	EMT, migration and invasion	136
m⁶A	Cervical cancer	IGF2BP3↑	SCD	/	Oncogene	Proliferation, migration and invasion, tumor size and lymphatic metastasis	137
m⁶A	AML	IGF2BP2↑	PRMT6	MFSD2A	Oncogene	Proliferation and apoptosis	138
m⁶A	Glioma	YTHDF2↑	LXRα and HIVEP2	/	Anti-oncogene	Proliferation, migration, invasion and tumorigenesis	139
m⁵C	OS	NSUN2↑	FABP5	/	Oncogene	Proliferation, migration and invasion, tumor size	141
m⁵C	GC	NSUN2↑	ORAI2	/	Oncogene	Adhesion, migration and invasion	142
m⁵C	PCa	NSUN5↑	ACC1	/	Oncogene	Proliferation, tumor size	143
m⁵C	HCC	NSUN2/YBX1↑	SREBP2	/	Oncogene	Proliferation, migration, and EMT	144
m⁵C	HCC	NSUN2↑	SOAT2	/	Oncogene	Proliferation, migration, and invasion	145
m¹A	HCC	TRMT6/TRMT61A↑	PPARδ	Hedgehog signaling pathway	Oncogene	Tumor size	147
m¹A	CRC	TRMT61A↑	tRNA	ACLY	Oncogene	Proliferation and tumor size	148

**Table 3:** The role of RNA methylation in amino acid metabolism reprogramming in cancer.

RNA methylation type	Cancer type	Methylase	Methylation target	Downstream effectors	Role of RNA methylation target	Types of metabolites	Reference
m⁶A	GC	FTO↑	circFAM192A	SCL7A5	oncogene	leucine	150
m⁶A	CRC	YTHDF1↑	GLS	/	oncogene	glutamine	151
m⁶A	CRC	YTHDF2↓	ATF4	mTOR	oncogene	glutamine	152
m⁶A	CRC	FTO↑	SCL7A11	GPX4	oncogene	cysteine, glutamic acid	153
m⁶A	LCA	METTL3↑	SCL7A5	/	oncogene	glutamine	154
m⁶A	LCA	IGF2BP2↑	SLC7A5	AKT/mTOR pathway	oncogene	methionine	155
m⁶A	BRCA	RBM15↑	PHGDH, PSAT1, PSPH, and SHMT2	/	oncogene	serine and glycine	156
m⁶A	CESC	METTL3↑	SLC38A1	/	oncogene	glutamine	157
m⁶A	UCEC	METTL14↑	PRMT3	/	oncogene	arginine	158
m⁶A	Melanoma	YTHDF3↑	LOXL3	/	oncogene	lysyl	159
m⁶A	AML	IGF2BP2↑	MYC, GPT2, and SLC1A5	/	oncogene	glutamine	160
m⁶A	AML	METTL16↑	BCAT1 and BCAT2	/	oncogene	Valine, leucine and isoleucine	161
m⁶A	ESCC	METTL3↑	NR4A2/IGF2BP2	/	oncogene	methionine	162
m⁶A	DLBCL	YTHDF2↑	ACER2	SphK/S1P/PI3K/AKT pathway	oncogene	ceramide	163
m⁵C	GC	NSUN2↑	lncRNA NR_033928	GLS	oncogene	glutamine	164
m⁵C	PAAD	ALYREF↑	JunD	SLC7A5/mTOR1	oncogene	large neutral amino acids	166
m⁵C	AML	NSUN2↑	PHGDH and SHMT2	/	oncogene	serine and glycine	167

**Table 4:** The role of RNA methylation in other metabolism reprogramming in cancer.

RNA methylation type	Cancer type	Methylase	Methylation target	Downstream effectors	Role of RNA methylation target	Phenotype	Metabolic type	Reference
m⁶A	GBM	METTL3↑	GPX4	/	Oncogene	Tumor size	Ferroptosis	170
m⁶A	CESC	METTL14↓	FTH1	PI3K/Akt signaling pathway	Oncogene	Chemoresistance	Ferroptosis	171
m⁶A	NSCLC	METTL14↑	GPX4	/	Oncogene	Proliferation	Ferroptosis	172
m⁶A	BRCA	METTL16↑	GPX4	/	Oncogene	Proliferation and tumor size	Ferroptosis	173
m⁶A	EC	HNRNPA2B1↑	FOXM1	LCN2	Oncogene	Proliferation, migration and invasion	Ferroptosis	174
m⁶A	BCa	WTAP↑	NRF2	/	Oncogene	Proliferation	Ferroptosis	175
m⁶A	GC	YTHDF2↑	CBS	ACSL4	Oncogene	Tumor size	Ferroptosis	176
m⁶A	GBM	METTL3↑	SLC7A11	/	Oncogene	Tumor size	Ferroptosis	177
m⁶A	THCA	FTO↓	SCL7A11	/	Oncogene	Proliferation, invasion and migration, and Ferroptosis	Ferroptosis	178
m⁶A	CRC	FTO↑	SCL7A11	GPX4	Oncogene	Tumor size	Ferroptosis	153
m⁶A	CRC	FTO↑ and YTHDF2↓	GPX4	/	Oncogene	Proliferation	Ferroptosis	179
m⁶A	CRC	METTL14↓ and YTHDF2↓	SLC7A11, SLC3A2, HOXA13, and GPX4	/	Oncogene	Apoptosis	Ferroptosis	180
m⁶A	HB	METTL3↑ and YTHDF2↑	LATS2	YAP1/ATF4/PSAT1	Anti-oncogene	Proliferation	Ferroptosis	181
m⁵C	EC	NSUN2↑	SLC7A11	/	Oncogene	Proliferation and tumor size	Ferroptosis	182
m⁵C	GC	NSUN2↑	GCLC	/	Oncogene	Cell viability	Ferroptosis	183
m⁵C	AML	NSUN2↑	FSP1	/	Oncogene	Proliferation	Ferroptosis	184
m⁵C	AML	ALKBH3↑	ATF4	SLC7A11, GPX4 and FTH1	Oncogene	Proliferation and apoptosis	Ferroptosis	185
m⁵C	HCC	NSUN2↑ and ALYREF↑	MALAT1	ELAVL1/SLC7A11	Oncogene	Proliferation and Chemoresistance	Ferroptosis	186
m⁵C	HCC	YBX1↑	RNF115	DHODH	Oncogene	Proliferation	Ferroptosis	187
m⁵C	NSCLC	NSUN2↑	NRF2	GPX4 and FTH1	Oncogene	Proliferation, migration, and invasion	Ferroptosis	188
m⁷G	OS	METTL1↓	pri-miR-26a	FTH1	Oncogene	Proliferation, migration, invasion and tumor growth	Ferroptosis	189
m⁶A	ccRCC	FTO↓	PGC-1α	/	Anti-oncogene	Cell growth and apoptosis	Mitochondrial metabolism	191
m⁶A	CRC	METTL3↑	miR-676, miR-483, and miR-877	ATP5I, ATP5G1, ATP5G3 and CYC1	Oncogene	Cell proliferation and apoptosis	Mitochondrial metabolism	192
m⁶A	CRC	METTL14↓	pri-miR-17	MFN2	Oncogene	Proliferation and apoptosis	Mitochondrial metabolism	193
m⁶A	GC	FTO↑	caveolin-1	/	Anti-oncogene	Proliferation, migration, and invasion	Mitochondrial metabolism	194
m⁶A	OC	IGF2BP1↑	FTH1	/	Oncogene	Proliferation and migration	Mitochondrial metabolism	195
m⁶A	OC	WTAP↑ and IGF2BP3↑	ULK1	/	Oncogene	Proliferation and migration	Mitochondrial metabolism	196
m⁶A	SCLC	METTL3↑	DCP2	Pink1-Parkin	Anti-oncogene	Proliferation	Mitochondrial metabolism	197
m⁵C	LCA	NSUN4↑	circERI3	/	Oncogene	Proliferation, cell viability, migration, cell cycle and apoptosis	Mitochondrial metabolism	198
m⁵C	LCA	NSUN2↑	circRREB1	HSPA8/PINK1/Parkin	Oncogene	Proliferation, cell viability, migration, cell cycle and apoptosis	Mitochondrial metabolism	199
m⁵C	B cell malignancies	YTHDF2↑	ATP	/	Oncogene	Proliferation	Mitochondrial metabolism	200
m⁶Am	RCC	PCIF1↑	LPP3	/	Oncogene	Proliferation and migration	Mitochondrial metabolism	201
m⁷G	GC	METTL1↑ and WDR4↑	SDHAF4	SDHA and SDHB	Oncogene	Proliferation, migration, invasion, colony formation, and anti-apoptotic abilities	Mitochondrial metabolism	202
m¹A	PCa	TRMT61A↑	p-PI3K, CPT1A and CPT1B	/	Oncogene	Colony formation ability, migration and invasion	Mitochondrial metabolism	203

**Table 5:** Landscape of m⁶A regulator inhibitors and clinical trial status

Target	Inhibitor	Cell type	Cancer type	Clinical trial status	Reference
METTL3	UZH1a	U2OS	Osteosarcoma	Preclinical	253
METTL3	UZH2	MOLM-13 and PC-3	AML and PCa	Preclinical	254
METTL3	STM2457	MOLM-13, THP-1, NOMO-1, EOL-1, KASUMI-1 and HL-60	AML	Preclinical	255
METTL3	STC-15	/	Advanced malignancies	Phase I Clinical Trial (NCT05584111 and NCT06975293)	256
FTO	Rhein	K562, KU812, MV4-11 and Kasumi-1	TKI-resistant leukemia	Preclinical	258
FTO	MO-I-500	SUM149-Luc	TNBC	Preclinical	259
FTO	Compound 12	HeLa cells	/	Preclinical	260
FTO	FB23-2	NB4, U937, MV4-11, and ML-2	AML	Preclinical	261
FTO	CS1 and CS2	U937, THP1 and MV4-11	AML	Preclinical	262
FTO	R-2HG	AML: U937, THP1, MV4-11, JURKAT, and HEL Glioblastoma: 8MGBA, A172, U87MG, GAMG, T98G, LN229, LN18, and DK-MG	leukemia and glioblastoma	Preclinical	263
FTO	Meclofenamic acid	HeLa cells	/	Preclinical	264
FTO	Meclofenamic acid	U87, U251 and A172	Glioma	Preclinical	265
					
ALKBH5	MV1035	U87-MG, H460 and A549	Glioblastoma	Preclinical	266
ALKBH5	ALK-04	CT26 and B16	CRC and Melanoma	Preclinical	267
ALKBH5	2-[(1-hydroxy-2-oxo-2-phenylethyl)sulfanyl]acetic acid (3) and 4-[268]-1,2-diazinane-3,6-dione (6)	HL-60, CCRF-CEM, Jurkat, K562 and A-172	leukemia and glioblastoma	Preclinical	268
YTHDF	Ebselen	PC-3	Prostate cancer	Preclinical	269
YTHDF2	DC-Y13-27	MC38, B16F1, B16-OVA and LLC	CRC and Melanoma	Preclinical	270
IGF2BP1	BTYNB	H1299, ES2, RKO, LKR-M-FL, LKR-M-GFP	Lung cancer and Melanoma	Preclinical	271

## Abbreviations

m⁶A: N6-methyladenosine
m⁶Am: N6:2'-O-dimethyladenosine
m⁵C: 5-methylcytosine
m⁷G: N7-methylguanidine
SAM: S-adenosylmethionine
snRNA: small nuclear RNA
rRNA: ribosomal RNA
FTO: fat mass and obesity-associated protein
ALKBH5: alpha-ketoglutarate-dependent dioxygenase alkB homolog 5
YTHDF: YT521-B homology domain family
IGF2BPs: insulin-like growth factor 2 mRNA-binding proteins
HNRNPs: heterogeneous nuclear ribonucleoproteins
eRNA: enhancer RNA
NSUN: NOL1/NOP2/SUN
mRNA: messenger RNA
miRNA: microRNA
lncRNA: long non-coding RNA
TETs: ten-eleven translocation proteins
ALYREF: ALY/REF export factor
YBX1: Y-box binding protein 1
tRNAs: transfer RNAs
m¹A: N1-methyladenosine methylation
TRMT6-TRMT61A: tRNA methyltransferase 6-tRNA methyltransferase 61A
TRMT10C: tRNA methyltransferase 10C
SDR5C1: Short-chain dehydrogenase/reductase family 5C member 1
WDR4: WD repeat domain 4
RNMT: RNA guanine-7 methyltransferase
WBSCR22: Williams-Beuren syndrome chromosome region 22
TRMT112: tRNA methyltransferase 112
eIF4E: eukaryotic initiation factor 4E
APC: adenomatous polyposis coli
PKM2: pyruvate kinase M2
EC: esophageal cancer
ESCC: esophageal squamous cell carcinoma
GC: gastric cancer
CCA: cholangiocarcinoma
PDAC: pancreatic ductal adenocarcinoma
6PGD: 6-phosphogluconate dehydrogenase
PPP: pentose phosphate pathway
HPV: human papilloma virus
HIF: hypoxia inducible factor
R-2HG: R-2-hydroxyglutaric acid
LUSC: lung squamous cell carcinoma
LUAD: lung adenocarcinoma
ICC: intrahepatic cholangiocarcinoma
ROS: reactive oxygen species
OXPHOS: oxidative phosphorylation
CRC: colorectal cancer
FABP: fatty acid-binding protein
cGAS-STING: cyclic GMP-AMP synthase
FASN: fatty acid synthase
RPL24: ribosomal protein L24
pNENs: pancreatic neuroendocrine neoplasms
EMT: epithelial mesenchymal transformation
CESC: cervical squamous cell carcinoma
LSCs: leukemia stem cells
GLUD1: glutamate dehydrogenase
GLS: glutaminase
GSH: glutathione
GSSG: oxidized glutathione
LNAAs: large neutral amino acids
PHGDH: phosphoglycerate dehydrogenase
SHMT2: serine hydroxy methyltransferase 2
FTH1: ferritin heavy chain
RCC: renal cell carcinoma
LPA: lysophosphatidic acid
ETC: electron transport chain
IFN-γ: interferon-gamma
AUC: area under the curve
2OG: 2-oxoglutarate
TKI: tyrosine kinase inhibitor
IDH1/2: isocitrate dehydrogenases 1 and 2
MDSCs: myeloid-derived suppressor cells
SSP: serine synthesis pathway
TME: tumor microenvironment
FAO: fatty acid oxidation

## References

[1] Hotchkiss RD (1948). The quantitative separation of purines, pyrimidines, and nucleosides by paper chromatography. The Journal of biological chemistry.

[2] Yang B, Wang JQ, Tan Y, Yuan R, Chen ZS, Zou C (2021). RNA methylation and cancer treatment. Pharmacological research.

[3] Dai X, Ren T, Zhang Y, Nan N (2021). Methylation multiplicity and its clinical values in cancer. Expert reviews in molecular medicine.

[4] Zhang H, Zhang L, Lin A, Xu C, Li Z, Liu K (2023). Algorithm for optimized mRNA design improves stability and immunogenicity. Nature.

[5] Siegel RL, Giaquinto AN, Jemal A (2024). Cancer statistics, 2024. CA: a cancer journal for clinicians.

[6] Bray F, Laversanne M, Sung H, Ferlay J, Siegel RL, Soerjomataram I (2024). Global cancer statistics 2022: GLOBOCAN estimates of incidence and mortality worldwide for 36 cancers in 185 countries. CA: a cancer journal for clinicians.

[7] Schmidt DR, Patel R, Kirsch DG, Lewis CA, Vander Heiden MG, Locasale JW (2021). Metabolomics in cancer research and emerging applications in clinical oncology. CA: a cancer journal for clinicians.

[8] You M, Xie Z, Zhang N, Zhang Y, Xiao D, Liu S (2023). Signaling pathways in cancer metabolism: mechanisms and therapeutic targets. Signal transduction and targeted therapy.

[9] Stine ZE, Schug ZT, Salvino JM, Dang CV (2022). Targeting cancer metabolism in the era of precision oncology. Nature reviews Drug discovery.

[10] Xiao Y, Yu TJ, Xu Y, Ding R, Wang YP, Jiang YZ (2023). Emerging therapies in cancer metabolism. Cell metabolism.

[11] Oerum S, Meynier V, Catala M, Tisné C (2021). A comprehensive review of m6A/m6Am RNA methyltransferase structures. Nucleic acids research.

[12] He L, Li H, Wu A, Peng Y, Shu G, Yin G (2019). Functions of N6-methyladenosine and its role in cancer. Molecular cancer.

[13] Ma S, Chen C, Ji X, Liu J, Zhou Q, Wang G (2019). The interplay between m6A RNA methylation and noncoding RNA in cancer. Journal of hematology & oncology.

[14] Lin S, Choe J, Du P, Triboulet R, Gregory RI (2016). The m(6)A Methyltransferase METTL3 Promotes Translation in Human Cancer Cells. Molecular cell.

[15] Sendinc E, Shi Y (2023). RNA m6A methylation across the transcriptome. Molecular cell.

[16] Tsujikawa K, Koike K, Kitae K, Shinkawa A, Arima H, Suzuki T (2007). Expression and sub-cellular localization of human ABH family molecules. Journal of cellular and molecular medicine.

[17] Cui L, Ma R, Cai J, Guo C, Chen Z, Yao L (2022). RNA modifications: importance in immune cell biology and related diseases. Signal transduction and targeted therapy.

[18] Dai XY, Shi L, Li Z, Yang HY, Wei JF, Ding Q (2021). Main N6-Methyladenosine Readers: YTH Family Proteins in Cancers. Frontiers in oncology.

[19] Yu D, Pan M, Li Y, Lu T, Wang Z, Liu C (2022). RNA N6-methyladenosine reader IGF2BP2 promotes lymphatic metastasis and epithelial-mesenchymal transition of head and neck squamous carcinoma cells via stabilizing slug mRNA in an m6A-dependent manner. Journal of experimental & clinical cancer research: CR.

[20] Trixl L, Lusser A (2019). The dynamic RNA modification 5-methylcytosine and its emerging role as an epitranscriptomic mark. Wiley interdisciplinary reviews RNA.

[21] Zhang Q, Sun X, Sun J, Lu J, Gao X, Shen K (2022). RNA m(5)C regulator-mediated modification patterns and the cross-talk between tumor microenvironment infiltration in gastric cancer. Frontiers in immunology.

[22] Li P, Huang D (2024). NSUN2-mediated RNA methylation: Molecular mechanisms and clinical relevance in cancer. Cellular signalling.

[23] Liao H, Gaur A, McConie H, Shekar A, Wang K, Chang JT (2022). Human NOP2/NSUN1 regulates ribosome biogenesis through non-catalytic complex formation with box C/D snoRNPs. Nucleic acids research.

[24] Nakano S, Suzuki T, Kawarada L, Iwata H, Asano K, Suzuki T (2016). NSUN3 methylase initiates 5-formylcytidine biogenesis in human mitochondrial tRNA(Met). Nature chemical biology.

[25] Metodiev MD, Spåhr H, Loguercio Polosa P, Meharg C, Becker C, Altmueller J (2014). NSUN4 is a dual function mitochondrial protein required for both methylation of 12S rRNA and coordination of mitoribosomal assembly. PLoS genetics.

[26] Liu RJ, Long T, Li J, Li H, Wang ED (2017). Structural basis for substrate binding and catalytic mechanism of a human RNA:m5C methyltransferase NSun6. Nucleic acids research.

[27] Ortiz-Barahona V, Soler M, Davalos V, García-Prieto CA, Janin M, Setien F (2023). Epigenetic inactivation of the 5-methylcytosine RNA methyltransferase NSUN7 is associated with clinical outcome and therapeutic vulnerability in liver cancer. Molecular cancer.

[28] Hu L, Lu J, Cheng J, Rao Q, Li Z, Hou H (2015). Structural insight into substrate preference for TET-mediated oxidation. Nature.

[29] Arguello AE, Li A, Sun X, Eggert TW, Mairhofer E, Kleiner RE (2022). Reactivity-dependent profiling of RNA 5-methylcytidine dioxygenases. Nature communications.

[30] Shi M, Zhang H, Wu X, He Z, Wang L, Yin S (2017). ALYREF mainly binds to the 5' and the 3' regions of the mRNA in vivo. Nucleic acids research.

[31] Yang Y, Wang L, Han X, Yang WL, Zhang M, Ma HL (2019). RNA 5-Methylcytosine Facilitates the Maternal-to-Zygotic Transition by Preventing Maternal mRNA Decay. Molecular cell.

[32] Zhang L, Li Y, Li L, Yao F, Cai M, Ye D (2025). Detection, molecular function and mechanisms of m5C in cancer. Clinical and translational medicine.

[33] Sharma S, Hartmann JD, Watzinger P, Klepper A, Peifer C, Kötter P (2018). A single N(1)-methyladenosine on the large ribosomal subunit rRNA impacts locally its structure and the translation of key metabolic enzymes. Scientific reports.

[34] Yang W, Meng J, Liu J, Ding B, Tan T, Wei Q (2020). The N(1)-Methyladenosine Methylome of Petunia mRNA. Plant physiology.

[35] Macon JB, Wolfenden R (1968). 1-Methyladenosine. Dimroth rearrangement and reversible reduction. Biochemistry.

[36] Ozanick S, Krecic A, Andersland J, Anderson JT (2005). The bipartite structure of the tRNA m1A58 methyltransferase from S. cerevisiae is conserved in humans. RNA (New York, NY).

[37] Wang M, Zhu Y, Wang C, Fan X, Jiang X, Ebrahimi M (2016). Crystal structure of the two-subunit tRNA m(1)A58 methyltransferase TRM6-TRM61 from Saccharomyces cerevisiae. Scientific reports.

[38] Vilardo E, Nachbagauer C, Buzet A, Taschner A, Holzmann J, Rossmanith W (2012). A subcomplex of human mitochondrial RNase P is a bifunctional methyltransferase-extensive moonlighting in mitochondrial tRNA biogenesis. Nucleic acids research.

[39] Chen Z, Qi M, Shen B, Luo G, Wu Y, Li J (2019). Transfer RNA demethylase ALKBH3 promotes cancer progression via induction of tRNA-derived small RNAs. Nucleic acids research.

[40] Wei J, Liu F, Lu Z, Fei Q, Ai Y, He PC (2018). Differential m(6)A, m(6)A(m), and m(1)A Demethylation Mediated by FTO in the Cell Nucleus and Cytoplasm. Molecular cell.

[41] Dai X, Wang T, Gonzalez G, Wang Y (2018). Identification of YTH Domain-Containing Proteins as the Readers for N1-Methyladenosine in RNA. Analytical chemistry.

[42] Zhang X, Zhu WY, Shen SY, Shen JH, Chen XD (2023). Biological roles of RNA m7G modification and its implications in cancer. Biology direct.

[43] Han M, Huang Q, Li X, Chen X, Zhu H, Pan Y (2024). M7G-related tumor immunity: novel insights of RNA modification and potential therapeutic targets. International journal of biological sciences.

[44] Luo Y, Yao Y, Wu P, Zi X, Sun N, He J (2022). The potential role of N(7)-methylguanosine (m7G) in cancer. Journal of hematology & oncology.

[45] Qiu L, Jing Q, Li Y, Han J (2023). RNA modification: mechanisms and therapeutic targets. Molecular biomedicine.

[46] Zhou W, Yi Y, Cao W, Zhong X, Chen L (2024). Functions of METTL1/WDR4 and QKI as m7G modification - related enzymes in digestive diseases. Frontiers in pharmacology.

[47] Mills JG, Hepburn LA, Cowling VH (2025). RNMT-dependent RNA cap methylation in health and disease. The Biochemical journal.

[48] Zorbas C, Nicolas E, Wacheul L, Huvelle E, Heurgué-Hamard V, Lafontaine DL (2015). The human 18S rRNA base methyltransferases DIMT1L and WBSCR22-TRMT112 but not rRNA modification are required for ribosome biogenesis. Molecular biology of the cell.

[49] Chen D, Gu X, Nurzat Y, Xu L, Li X, Wu L (2024). Writers, readers, and erasers RNA modifications and drug resistance in cancer. Molecular cancer.

[50] Boris-Lawrie K, Liebau J, Hayir A, Heng X (2025). Emerging Roles of m7G-Cap Hypermethylation and Nuclear Cap-Binding Proteins in Bypassing Suppression of eIF4E-Dependent Translation. Viruses.

[51] Wang W, Shao F, Yang X, Wang J, Zhu R, Yang Y (2021). METTL3 promotes tumour development by decreasing APC expression mediated by APC mRNA N(6)-methyladenosine-dependent YTHDF binding. Nature communications.

[52] Huang T, You Q, Liu J, Shen X, Huang D, Tao X (2025). WTAP Mediated m6A Modification Stabilizes PDIA3P1 and Promotes Tumor Progression Driven by Histone Lactylation in Esophageal Squamous Cell Carcinoma. Advanced science (Weinheim, Baden-Wurttemberg, Germany).

[53] Wang Q, Chen C, Ding Q, Zhao Y, Wang Z, Chen J (2020). METTL3-mediated m(6)A modification of HDGF mRNA promotes gastric cancer progression and has prognostic significance. Gut.

[54] Xu W, Lai Y, Pan Y, Tan M, Ma Y, Sheng H (2022). m6A RNA methylation-mediated NDUFA4 promotes cell proliferation and metabolism in gastric cancer. Cell death & disease.

[55] Lin JX, Lian NZ, Gao YX, Zheng QL, Yang YH, Ma YB (2022). m6A methylation mediates LHPP acetylation as a tumour aerobic glycolysis suppressor to improve the prognosis of gastric cancer. Cell death & disease.

[56] Yu H, Zhao K, Zeng H, Li Z, Chen K, Zhang Z (2021). N(6)-methyladenosine (m(6)A) methyltransferase WTAP accelerates the Warburg effect of gastric cancer through regulating HK2 stability. Biomedicine & pharmacotherapy = Biomedecine & pharmacotherapie.

[57] Yang D, Chang S, Li F, Ma M, Yang J, Lv X (2021). m(6) A transferase KIAA1429-stabilized LINC00958 accelerates gastric cancer aerobic glycolysis through targeting GLUT1. IUBMB life.

[58] Xu TP, Yu T, Xie MY, Fang Y, Xu TT, Pan YT (2023). LOC101929709 promotes gastric cancer progression by aiding LIN28B to stabilize c-MYC mRNA. Gastric cancer: official journal of the International Gastric Cancer Association and the Japanese Gastric Cancer Association.

[59] Shen C, Xuan B, Yan T, Ma Y, Xu P, Tian X (2020). m(6)A-dependent glycolysis enhances colorectal cancer progression. Molecular cancer.

[60] Chen H, Gao S, Liu W, Wong CC, Wu J, Wu J (2021). RNA N(6)-Methyladenosine Methyltransferase METTL3 Facilitates Colorectal Cancer by Activating the m(6)A-GLUT1-mTORC1 Axis and Is a Therapeutic Target. Gastroenterology.

[61] Zhang K, Zhang T, Yang Y, Tu W, Huang H, Wang Y (2022). N(6)-methyladenosine-mediated LDHA induction potentiates chemoresistance of colorectal cancer cells through metabolic reprogramming. Theranostics.

[62] Zheng Y, Wang Y, Liu Y, Xie L, Ge J, Yu G (2021). N6-Methyladenosine Modification of PTTG3P Contributes to Colorectal Cancer Proliferation via YAP1. Frontiers in oncology.

[63] Hou Y, Zhang X, Yao H, Hou L, Zhang Q, Tao E (2023). METTL14 modulates glycolysis to inhibit colorectal tumorigenesis in p53-wild-type cells. EMBO reports.

[64] Li Y, He L, Wang Y, Tan Y, Zhang F (2022). N(6)-methyladenosine methyltransferase KIAA1429 elevates colorectal cancer aerobic glycolysis via HK2-dependent manner. Bioengineered.

[65] Chen B, Hong Y, Gui R, Zheng H, Tian S, Zhai X (2022). N6-methyladenosine modification of circ_0003215 suppresses the pentose phosphate pathway and malignancy of colorectal cancer through the miR-663b/DLG4/G6PD axis. Cell death & disease.

[66] Zhang Y, Tian X, Bai Y, Liu X, Zhu J, Zhang L (2022). WTAP mediates FOXP3 mRNA stability to promote SMARCE1 expression and augment glycolysis in colon adenocarcinoma. Mammalian genome: official journal of the International Mammalian Genome Society.

[67] Li Z, Peng Y, Li J, Chen Z, Chen F, Tu J (2020). N(6)-methyladenosine regulates glycolysis of cancer cells through PDK4. Nature communications.

[68] Du L, Li Y, Kang M, Feng M, Ren Y, Dai H (2021). USP48 Is Upregulated by Mettl14 to Attenuate Hepatocellular Carcinoma via Regulating SIRT6 Stabilization. Cancer research.

[69] Wang Q, Xie H, Peng H, Yan J, Han L, Ye G (2021). ZC3H13 Inhibits the Progression of Hepatocellular Carcinoma through m(6)A-PKM2-Mediated Glycolysis and Enhances Chemosensitivity. Journal of oncology.

[70] Li J, Zhu L, Shi Y, Liu J, Lin L, Chen X (2019). m6A demethylase FTO promotes hepatocellular carcinoma tumorigenesis via mediating PKM2 demethylation. American journal of translational research.

[71] Zhao L, Kang M, Liu X, Wang Z, Wang Y, Chen H (2022). UBR7 inhibits HCC tumorigenesis by targeting Keap1/Nrf2/Bach1/HK2 and glycolysis. Journal of experimental & clinical cancer research: CR.

[72] Zhou R, Ni W, Qin C, Zhou Y, Li Y, Huo J (2022). A functional loop between YTH domain family protein YTHDF3 mediated m(6)A modification and phosphofructokinase PFKL in glycolysis of hepatocellular carcinoma. Journal of experimental & clinical cancer research: CR.

[73] Ye Y, Wang M, Wang G, Mai Z, Zhou B, Han Y (2023). lncRNA miR4458HG modulates hepatocellular carcinoma progression by activating m6A-dependent glycolysis and promoting the polarization of tumor-associated macrophages. Cellular and molecular life sciences: CMLS.

[74] Cai J, Cui Z, Zhou J, Zhang B, Lu R, Ding Y (2022). METTL3 promotes glycolysis and cholangiocarcinoma progression by mediating the m6A modification of AKR1B10. Cancer cell international.

[75] Li F, He C, Yao H, Zhao Y, Ye X, Zhou S (2023). Glutamate from nerve cells promotes perineural invasion in pancreatic cancer by regulating tumor glycolysis through HK2 mRNA-m6A modification. Pharmacological research.

[76] Hu Y, Tang J, Xu F, Chen J, Zeng Z, Han S (2022). A reciprocal feedback between N6-methyladenosine reader YTHDF3 and lncRNA DICER1-AS1 promotes glycolysis of pancreatic cancer through inhibiting maturation of miR-5586-5p. Journal of experimental & clinical cancer research: CR.

[77] Sheng H, Li Z, Su S, Sun W, Zhang X, Li L (2020). YTH domain family 2 promotes lung cancer cell growth by facilitating 6-phosphogluconate dehydrogenase mRNA translation. Carcinogenesis.

[78] Ma L, Xue X, Zhang X, Yu K, Xu X, Tian X (2022). The essential roles of m(6)A RNA modification to stimulate ENO1-dependent glycolysis and tumorigenesis in lung adenocarcinoma. Journal of experimental & clinical cancer research: CR.

[79] Yang X, Shao F, Guo D, Wang W, Wang J, Zhu R (2021). WNT/β-catenin-suppressed FTO expression increases m(6)A of c-Myc mRNA to promote tumor cell glycolysis and tumorigenesis. Cell death & disease.

[80] Zhang Q, Zhang Y, Chen H, Sun LN, Zhang B, Yue DS (2022). METTL3-induced DLGAP1-AS2 promotes non-small cell lung cancer tumorigenesis through m(6)A/c-Myc-dependent aerobic glycolysis. Cell cycle (Georgetown, Tex).

[81] Cui Y, Liu J, Liu L, Ma X, Gui Y, Liu H (2023). m(6)A-modified circFOXK2 targets GLUT1 to accelerate oral squamous cell carcinoma aerobic glycolysis. Cancer gene therapy.

[82] Xu K, Dai X, Wu J, Wen K (2022). N(6)-methyladenosine (m(6)A) reader IGF2BP2 stabilizes HK2 stability to accelerate the Warburg effect of oral squamous cell carcinoma progression. Journal of cancer research and clinical oncology.

[83] Xu Y, Song M, Hong Z, Chen W, Zhang Q, Zhou J (2023). The N6-methyladenosine METTL3 regulates tumorigenesis and glycolysis by mediating m6A methylation of the tumor suppressor LATS1 in breast cancer. Journal of experimental & clinical cancer research: CR.

[84] Ou B, Liu Y, Yang X, Xu X, Yan Y, Zhang J (2021). C5aR1-positive neutrophils promote breast cancer glycolysis through WTAP-dependent m6A methylation of ENO1. Cell death & disease.

[85] Liu H, Lyu H, Jiang G, Chen D, Ruan S, Liu S (2022). ALKBH5-Mediated m6A Demethylation of GLUT4 mRNA Promotes Glycolysis and Resistance to HER2-Targeted Therapy in Breast Cancer. Cancer research.

[86] Hu C, Liu T, Han C, Xuan Y, Jiang D, Sun Y (2022). HPV E6/E7 promotes aerobic glycolysis in cervical cancer by regulating IGF2BP2 to stabilize m(6)A-MYC expression. International journal of biological sciences.

[87] Liu C, Li Y, Dong C, Qu L, Zuo Y (2022). E6E7 regulates the HK2 expression in cervical cancer via GSK3β/FTO signal. Archives of biochemistry and biophysics.

[88] Lyu Y, Zhang Y, Wang Y, Luo Y, Ding H, Li P (2022). HIF-1α Regulated WTAP Overexpression Promoting the Warburg Effect of Ovarian Cancer by m6A-Dependent Manner. Journal of immunology research.

[89] Ji X, Lv C, Huang J, Dong W, Sun W, Zhang H (2023). ALKBH5-induced circular RNA NRIP1 promotes glycolysis in thyroid cancer cells by targeting PKM2. Cancer science.

[90] Huang J, Sun W, Wang Z, Lv C, Zhang T, Zhang D (2022). FTO suppresses glycolysis and growth of papillary thyroid cancer via decreasing stability of APOE mRNA in an N6-methyladenosine-dependent manner. Journal of experimental & clinical cancer research: CR.

[91] Liu Z, Chen Y, Wang L, Ji S (2021). ALKBH5 Promotes the Proliferation of Glioma Cells via Enhancing the mRNA Stability of G6PD. Neurochemical research.

[92] Li XD, Wang MJ, Zheng JL, Wu YH, Wang X, Jiang XB (2021). Long noncoding RNA just proximal to X-inactive specific transcript facilitates aerobic glycolysis and temozolomide chemoresistance by promoting stability of PDK1 mRNA in an m6A-dependent manner in glioblastoma multiforme cells. Cancer science.

[93] Qing Y, Dong L, Gao L, Li C, Li Y, Han L (2021). R-2-hydroxyglutarate attenuates aerobic glycolysis in leukemia by targeting the FTO/m(6)A/PFKP/LDHB axis. Molecular cell.

[94] Yang F, Liu Y, Xiao J, Li B, Chen Y, Hu A (2023). Circ-CTNNB1 drives aerobic glycolysis and osteosarcoma progression via m6A modification through interacting with RBM15. Cell proliferation.

[95] Yuan B, Zhou J (2023). N(6)-methyladenosine (m(6)A) reader IGF2BP1 facilitates clear-cell renal cell carcinoma aerobic glycolysis. PeerJ.

[96] Zhang C, Chen L, Liu Y, Huang J, Liu A, Xu Y (2021). Downregulated METTL14 accumulates BPTF that reinforces super-enhancers and distal lung metastasis via glycolytic reprogramming in renal cell carcinoma. Theranostics.

[97] Han H, Fan G, Song S, Jiang Y, Qian C, Zhang W (2021). piRNA-30473 contributes to tumorigenesis and poor prognosis by regulating m6A RNA methylation in DLBCL. Blood.

[98] Wang W, Ding Y, Zhao H, Wang S, Huang J, Sun L (2025). NSUN2-tRNA(Val-CAC)-axis-regulated codon-biased translation drives triple-negative breast cancer glycolysis and progression. Cellular & molecular biology letters.

[99] Li H, Liang Y, Tang J, Luo H, Wang Y (2025). YBX1 Enhances the Stability of TM4SF1 in an m5C-Dependent Manner to Promote Bladder Cancer Proliferation and Glycolysis. Combinatorial chemistry & high throughput screening.

[100] Wang JZ, Zhu W, Han J, Yang X, Zhou R, Lu HC (2021). The role of the HIF-1α/ALYREF/PKM2 axis in glycolysis and tumorigenesis of bladder cancer. Cancer communications (London, England).

[101] Yu T, Zhang Q, Yu SK, Nie FQ, Zhang ML, Wang Q (2023). THOC3 interacts with YBX1 to promote lung squamous cell carcinoma progression through PFKFB4 mRNA modification. Cell death & disease.

[102] Zhang RK, Li Y, Sun FL, Zhou ZH, Xie YX, Liu WJ (2024). RNA methyltransferase NSUN2-mediated m5C methylation promotes Cr(VI)-induced malignant transformation and lung cancer by accelerating metabolism reprogramming. Environment international.

[103] Liu S, Xu B, Zhao J (2025). NSUN2-mediated m5C modification of PGK1 mRNA promotes cell growth, invasion, stemness and glycolysis in gastric cancer. Cell cycle (Georgetown, Tex).

[104] Chen B, Deng Y, Hong Y, Fan L, Zhai X, Hu H (2024). Metabolic Recoding of NSUN2-Mediated m(5)C Modification Promotes the Progression of Colorectal Cancer via the NSUN2/YBX1/m(5)C-ENO1 Positive Feedback Loop. Advanced science (Weinheim, Baden-Wurttemberg, Germany).

[105] Zhang H, Zhai X, Liu Y, Xia Z, Xia T, Du G (2023). NOP2-mediated m5C Modification of c-Myc in an EIF3A-Dependent Manner to Reprogram Glucose Metabolism and Promote Hepatocellular Carcinoma Progression. Research (Washington, DC).

[106] Qi Q, Zhong R, Huang Y, Tang Y, Zhang XW, Liu C (2025). The RNA M5C methyltransferase NSUN2 promotes progression of hepatocellular carcinoma by enhancing PKM2-mediated glycolysis. Cell death & disease.

[107] Wang X, Duan W, Ma Z, Wen H, Mao X, Liu C (2025). ETV4/ALYREF-mediated glycolytic metabolism through PKM2 enhances resistance to ferroptosis and promotes the development of intrahepatic cholangiocarcinoma. Cancer & metabolism.

[108] Chen T, Xu ZG, Luo J, Manne RK, Wang Z, Hsu CC (2023). NSUN2 is a glucose sensor suppressing cGAS/STING to maintain tumorigenesis and immunotherapy resistance. Cell metabolism.

[109] Wang K, Kong F, Han X, Zhi Y, Wang H, Ren C (2025). Integrative multi-omics reveal NSUN2 facilitates glycolysis and histone lactylation-driven immune evasion in renal carcinoma. Genes and immunity.

[110] Tian Y, Liu J, Sun L, Wang X (2025). ALYREF regulates the m5C modification and stability of BIRC5 mRNA to promote ovarian cancer progression. Pathology, research and practice.

[111] Guan J, Lu L, Jiang Y (2025). NSUN2 contributes to the RB malignant progression and Glycolysis by mediating the m5C methylation modification of HKDC1. Journal of bioenergetics and biomembranes.

[112] Chen J, Zhou Q, Li S, Ling R, Zhao Y, Chen D (2024). Metabolic reprogramming driven by METTL1-mediated tRNA m7G modification promotes acquired anlotinib resistance in oral squamous cell carcinoma. Translational research: the journal of laboratory and clinical medicine.

[113] Xiao C, Hou G, Wang C, Huang Y, Liu Z (2025). METTL1 mediates m7G modification of PFKFB3 mRNA to promote radioresistance in esophageal cancer by affecting glycolytic metabolism. Pathology, research and practice.

[114] Zhao Q, Li X, Wu J, Zhang R, Chen S, Cai D (2024). TRMT10C-mediated m7G modification of circFAM126A inhibits lung cancer growth by regulating cellular glycolysis. Cell biology and toxicology.

[115] Liu L, Li X, Hu X, Zhai D, Cao T, Liu L (2025). POU4F1 Promotes the Primary Resistance of Melanoma to Anti-PD-1 Therapy by Regulating Glycolysis Through METTL1-Mediated m7G Methylation of PKM2. Molecular carcinogenesis.

[116] Wang F, Yang C, Zheng F, Yan Y, Li G, Feng Y (2024). METTL1 mediates PKM m7G modification to regulate CD155 expression and promote immune evasion in colorectal cancer. Journal of translational medicine.

[117] Wu Y, Chen Z, Xie G, Zhang H, Wang Z, Zhou J (2022). RNA m(1)A methylation regulates glycolysis of cancer cells through modulating ATP5D. Proceedings of the National Academy of Sciences of the United States of America.

[118] Deng Y, Chen Z, Chen P, Xiong Y, Zhang C, Wu Q (2025). ALKBH3-regulated m(1)A of ALDOA potentiates glycolysis and doxorubicin resistance of triple negative breast cancer cells. Acta pharmaceutica Sinica B.

[119] Yoon H, Shaw JL, Haigis MC, Greka A (2021). Lipid metabolism in sickness and in health: Emerging regulators of lipotoxicity. Molecular cell.

[120] Guo H, Wang B, Xu K, Nie L, Fu Y, Wang Z (2020). m(6)A Reader HNRNPA2B1 Promotes Esophageal Cancer Progression via Up-Regulation of ACLY and ACC1. Frontiers in oncology.

[121] Duan X, Yang L, Wang L, Liu Q, Zhang K, Liu S (2022). m6A demethylase FTO promotes tumor progression via regulation of lipid metabolism in esophageal cancer. Cell & bioscience.

[122] Jia Y, Yan Q, Zheng Y, Li L, Zhang B, Chang Z (2022). Long non-coding RNA NEAT1 mediated RPRD1B stability facilitates fatty acid metabolism and lymph node metastasis via c-Jun/c-Fos/SREBP1 axis in gastric cancer. Journal of experimental & clinical cancer research: CR.

[123] Cai X, Li X, Zhang M, Dong Z, Weng Y, Yu W (2025). RBM15 promotes lipogenesis and malignancy in gastric cancer by regulating N6-Methyladenosine modification of ACLY mRNA in an IGF2BP2-dependent manner. Biochimica et biophysica acta Molecular and cell biology of lipids.

[124] Chen B, Hong Y, Zhai X, Deng Y, Hu H, Tian S (2023). m6A and m5C modification of GPX4 facilitates anticancer immunity via STING activation. Cell death & disease.

[125] Ye M, Hu C, Chen T, Yu P, Chen J, Lu F (2023). FABP5 suppresses colorectal cancer progression via mTOR-mediated autophagy by decreasing FASN expression. International journal of biological sciences.

[126] Zhang Q, Du Z, Zhou W, Li W, Yang Q, Yu H (2024). ZDHHC1 downregulates LIPG and inhibits colorectal cancer growth via IGF2BP1 Palmitoylation. Cancer gene therapy.

[127] Guo W, Zhang C, Feng P, Li M, Wang X, Xia Y (2021). M6A methylation of DEGS2, a key ceramide-synthesizing enzyme, is involved in colorectal cancer progression through ceramide synthesis. Oncogene.

[128] Yang Y, Cai J, Yang X, Wang K, Sun K, Yang Z (2022). Dysregulated m6A modification promotes lipogenesis and development of non-alcoholic fatty liver disease and hepatocellular carcinoma. Molecular therapy: the journal of the American Society of Gene Therapy.

[129] Peng H, Chen B, Wei W, Guo S, Han H, Yang C (2022). N(6)-methyladenosine (m(6)A) in 18S rRNA promotes fatty acid metabolism and oncogenic transformation. Nature metabolism.

[130] Tong H, Yu X, Zhou D, Shen Z, Chen J, Si Y (2024). CircEZH2 promotes gallbladder cancer progression and lipid metabolism reprogramming through the miR-556-5p/SCD1 axis. iScience.

[131] Chen J, Ye M, Bai J, Gong Z, Yan L, Gu D (2023). ALKBH5 enhances lipid metabolism reprogramming by increasing stability of FABP5 to promote pancreatic neuroendocrine neoplasms progression in an m6A-IGF2BP2-dependent manner. Journal of translational medicine.

[132] Liu P, Fan B, Othmane B, Hu J, Li H, Cui Y (2022). m(6)A-induced lncDBET promotes the malignant progression of bladder cancer through FABP5-mediated lipid metabolism. Theranostics.

[133] Shi J, Miao D, Lv Q, Wang K, Wang Q, Liang H (2023). The m6A modification-mediated OGDHL exerts a tumor suppressor role in ccRCC by downregulating FASN to inhibit lipid synthesis and ERK signaling. Cell death & disease.

[134] Shi J, Lv Q, Miao D, Xiong Z, Wei Z, Wu S (2024). HIF2α Promotes Cancer Metastasis through TCF7L2-Dependent Fatty Acid Synthesis in ccRCC. Research (Washington, DC).

[135] Li Q, Wang Y, Meng X, Wang W, Duan F, Chen S (2024). METTL16 inhibits papillary thyroid cancer tumorigenicity through m(6)A/YTHDC2/SCD1-regulated lipid metabolism. Cellular and molecular life sciences: CMLS.

[136] Zhen L, Pan W (2023). ALKBH5 inhibits the SIRT3/ACC1 axis to regulate fatty acid metabolism via an m6A-IGF2BP1-dependent manner in cervical squamous cell carcinoma. Clinical and experimental pharmacology & physiology.

[137] Han C, Hu C, Liu T, Sun Y, Hu F, He Y (2024). IGF2BP3 enhances lipid metabolism in cervical cancer by upregulating the expression of SCD. Cell death & disease.

[138] Cheng Y, Gao Z, Zhang T, Wang Y, Xie X, Han G (2023). Decoding m(6)A RNA methylome identifies PRMT6-regulated lipid transport promoting AML stem cell maintenance. Cell stem cell.

[139] Fang R, Chen X, Zhang S, Shi H, Ye Y, Shi H (2021). EGFR/SRC/ERK-stabilized YTHDF2 promotes cholesterol dysregulation and invasive growth of glioblastoma. Nature communications.

[140] Snaebjornsson MT, Janaki-Raman S, Schulze A (2020). Greasing the Wheels of the Cancer Machine: The Role of Lipid Metabolism in Cancer. Cell metabolism.

[141] Yang M, Wei R, Zhang S, Hu S, Liang X, Yang Z (2023). NSUN2 promotes osteosarcoma progression by enhancing the stability of FABP5 mRNA via m(5)C methylation. Cell death & disease.

[142] Liu K, Xu P, Lv J, Ge H, Yan Z, Huang S (2023). Peritoneal high-fat environment promotes peritoneal metastasis of gastric cancer cells through activation of NSUN2-mediated ORAI2 m5C modification. Oncogene.

[143] Zhang Y, Chen XN, Zhang H, Wen JK, Gao HT, Shi B (2023). CDK13 promotes lipid deposition and prostate cancer progression by stimulating NSUN5-mediated m5C modification of ACC1 mRNA. Cell death and differentiation.

[144] Li R, Li S, Shen L, Li J, Zhang D, Yu J (2024). LINC00618 facilitates growth and metastasis of hepatocellular carcinoma via elevating cholesterol synthesis by promoting NSUN2-mediated SREBP2 m5C modification. Ecotoxicology and environmental safety.

[145] Jiang J, Liu F, Cui D, Xu C, Chi J, Yan T (2025). Novel molecular mechanisms of immune evasion in hepatocellular carcinoma: NSUN2-mediated increase of SOAT2 RNA methylation. Cancer communications (London, England).

[146] Su Z, Monshaugen I, Wilson B, Wang F, Klungland A, Ougland R (2022). TRMT6/61A-dependent base methylation of tRNA-derived fragments regulates gene-silencing activity and the unfolded protein response in bladder cancer. Nature communications.

[147] Wang Y, Wang J, Li X, Xiong X, Wang J, Zhou Z (2021). N(1)-methyladenosine methylation in tRNA drives liver tumourigenesis by regulating cholesterol metabolism. Nature communications.

[148] Miao S, Li H, Song X, Liu Y, Wang G, Kan C (2025). tRNA m1A modification regulates cholesterol biosynthesis to promote antitumor immunity of CD8+ T cells. The Journal of experimental medicine.

[149] Cluntun AA, Lukey MJ, Cerione RA, Locasale JW (2017). Glutamine Metabolism in Cancer: Understanding the Heterogeneity. Trends in cancer.

[150] Wu X, Fang Y, Gu Y, Shen H, Xu Y, Xu T (2024). Fat mass and obesity-associated protein (FTO) mediated m(6)A modification of circFAM192A promoted gastric cancer proliferation by suppressing SLC7A5 decay. Molecular biomedicine.

[151] Chen P, Liu XQ, Lin X, Gao LY, Zhang S, Huang X (2021). Targeting YTHDF1 effectively re-sensitizes cisplatin-resistant colon cancer cells by modulating GLS-mediated glutamine metabolism. Molecular therapy oncolytics.

[152] Han S, Zhu L, Zhu Y, Meng Y, Li J, Song P (2021). Targeting ATF4-dependent pro-survival autophagy to synergize glutaminolysis inhibition. Theranostics.

[153] Qiao Y, Su M, Zhao H, Liu H, Wang C, Dai X (2024). Targeting FTO induces colorectal cancer ferroptotic cell death by decreasing SLC7A11/GPX4 expression. Journal of experimental & clinical cancer research: CR.

[154] Fan Y, Yu Y (2024). Cancer-associated fibroblasts-derived exosomal METTL3 promotes the proliferation, invasion, stemness and glutaminolysis in non-small cell lung cancer cells by eliciting SLC7A5 m6A modification. Human cell.

[155] Zhou Z, Zhang B, Deng Y, Deng S, Li J, Wei W (2024). FBW7/GSK3β mediated degradation of IGF2BP2 inhibits IGF2BP2-SLC7A5 positive feedback loop and radioresistance in lung cancer. Journal of experimental & clinical cancer research: CR.

[156] Park SH, Ju JS, Woo H, Yun HJ, Lee SB, Kim SH (2024). The m(6)A writer RBM15 drives the growth of triple-negative breast cancer cells through the stimulation of serine and glycine metabolism. Experimental & molecular medicine.

[157] Liu HT, Zhao Y, Wang HC, Liu QL (2024). METTL3-mediated m(6)A methylation of SLC38A1 stimulates cervical cancer growth. Biochemical and biophysical research communications.

[158] Wang Y, Wang C, Guan X, Ma Y, Zhang S, Li F (2023). PRMT3-Mediated Arginine Methylation of METTL14 Promotes Malignant Progression and Treatment Resistance in Endometrial Carcinoma. Advanced science (Weinheim, Baden-Wurttemberg, Germany).

[159] Shi HZ, Xiong JS, Gan L, Zhang Y, Zhang CC, Kong YQ (2022). N6-methyladenosine reader YTHDF3 regulates melanoma metastasis via its 'executor'LOXL3. Clinical and translational medicine.

[160] Weng H, Huang F, Yu Z, Chen Z, Prince E, Kang Y (2022). The m(6)A reader IGF2BP2 regulates glutamine metabolism and represents a therapeutic target in acute myeloid leukemia. Cancer cell.

[161] Han L, Dong L, Leung K, Zhao Z, Li Y, Gao L (2023). METTL16 drives leukemogenesis and leukemia stem cell self-renewal by reprogramming BCAA metabolism. Cell stem cell.

[162] Jin X, Liu L, Liu D, Wu J, Wang C, Wang S (2024). Unveiling the methionine cycle: a key metabolic signature and NR4A2 as a methionine-responsive oncogene in esophageal squamous cell carcinoma. Cell death and differentiation.

[163] Chen X, Lu T, Ding M, Cai Y, Yu Z, Zhou X (2024). Targeting YTHDF2 inhibits tumorigenesis of diffuse large B-cell lymphoma through ACER2-mediated ceramide catabolism. Journal of advanced research.

[164] Fang L, Huang H, Lv J, Chen Z, Lu C, Jiang T (2023). m5C-methylated lncRNA NR_033928 promotes gastric cancer proliferation by stabilizing GLS mRNA to promote glutamine metabolism reprogramming. Cell death & disease.

[165] Liu Y, Xu W, Li M, Yang Y, Sun D, Chen L (2023). The regulatory mechanisms and inhibitors of isocitrate dehydrogenase 1 in cancer. Acta pharmaceutica Sinica B.

[166] Meng Q, Xie Y, Sun K, He L, Wu H, Zhang Q (2024). ALYREF-JunD-SLC7A5 axis promotes pancreatic ductal adenocarcinoma progression through epitranscriptome-metabolism reprogramming and immune evasion. Cell death discovery.

[167] Li S, Liu Y, Wu X, Pan M, Zhao H, Hong Y (2025). The m((5))C methyltransferase NSUN2 promotes progression of acute myeloid leukemia by regulating serine metabolism. Cell reports.

[168] Li X, Zhang HS (2024). Amino acid metabolism, redox balance and epigenetic regulation in cancer. The FEBS journal.

[169] Liu Y, Wan Y, Jiang Y, Zhang L, Cheng W (2023). GPX4: The hub of lipid oxidation, ferroptosis, disease and treatment. Biochimica et biophysica acta Reviews on cancer.

[170] Meng X, Wang Z, Yang Q, Liu Y, Gao Y, Chen H (2024). Intracellular C5aR1 inhibits ferroptosis in glioblastoma through METTL3-dependent m6A methylation of GPX4. Cell death & disease.

[171] Li L, Zeng J, He S, Yang Y, Wang C (2024). METTL14 decreases FTH1 mRNA stability via m6A methylation to promote sorafenib-induced ferroptosis of cervical cancer. Cancer biology & therapy.

[172] Lou Y, Huang K, Xu B, Chen X (2024). METTL14 plays an oncogenic role in NSCLC by modulating ferroptosis and the m6A modification of GPX4. Archives of physiology and biochemistry.

[173] Ye F, Wu J, Zhang F (2023). METTL16 epigenetically enhances GPX4 expression via m6A modification to promote breast cancer progression by inhibiting ferroptosis. Biochemical and biophysical research communications.

[174] Jiang J, Zhu J, Qiu P, Ni J, Zhu W, Wang X (2023). HNRNPA2B1-mediated m6A modification of FOXM1 promotes drug resistance and inhibits ferroptosis in endometrial cancer via regulation of LCN2. Functional & integrative genomics.

[175] Wang K, Wang G, Li G, Zhang W, Wang Y, Lin X (2023). m6A writer WTAP targets NRF2 to accelerate bladder cancer malignancy via m6A-dependent ferroptosis regulation. Apoptosis: an international journal on programmed cell death.

[176] Yang H, Hu Y, Weng M, Liu X, Wan P, Hu Y (2022). Hypoxia inducible lncRNA-CBSLR modulates ferroptosis through m6A-YTHDF2-dependent modulation of CBS in gastric cancer. Journal of advanced research.

[177] Sun S, Gao T, Pang B, Su X, Guo C, Zhang R (2022). RNA binding protein NKAP protects glioblastoma cells from ferroptosis by promoting SLC7A11 mRNA splicing in an m(6)A-dependent manner. Cell death & disease.

[178] Ji FH, Fu XH, Li GQ, He Q, Qiu XG (2022). FTO Prevents Thyroid Cancer Progression by SLC7A11 m6A Methylation in a Ferroptosis-Dependent Manner. Frontiers in endocrinology.

[179] Zhang G, Mi W, Wang C, Li J, Zhang Y, Liu N (2023). Targeting AKT induced Ferroptosis through FTO/YTHDF2-dependent GPX4 m6A methylation up-regulating and degradating in colorectal cancer. Cell death discovery.

[180] Wang F, Sun Z, Zhang Q, Yang H, Yang G, Yang Q (2023). Curdione induces ferroptosis mediated by m6A methylation via METTL14 and YTHDF2 in colorectal cancer. Chinese medicine.

[181] Zhu G, Xie Y, Bian Z, Ma J, Zhen N, Chen T (2024). N6-methyladenosine modification of LATS2 promotes hepatoblastoma progression by inhibiting ferroptosis through the YAP1/ATF4/PSAT1 axis. International journal of biological sciences.

[182] Chen SJ, Zhang J, Zhou T, Rao SS, Li Q, Xiao LY (2024). Epigenetically upregulated NSUN2 confers ferroptosis resistance in endometrial cancer via m(5)C modification of SLC7A11 mRNA. Redox biology.

[183] Niu K, Chen Z, Li M, Ma G, Deng Y, Zhang J (2025). NSUN2 lactylation drives cancer cell resistance to ferroptosis through enhancing GCLC-dependent glutathione synthesis. Redox biology.

[184] Ye W, Zhao Y, Zhou Y, Huang J, He X, Ma Z (2025). NSUN2-mediated cytosine-5 methylation of FSP1 protects acute myeloid leukemia cells from ferroptosis. Molecular cancer.

[185] Liu X, Pan X (2025). ALKBH3-mediated m1A demethylation promotes the malignant progression of acute myeloid leukemia by regulating ferroptosis through the upregulation of ATF4 expression. Hematology (Amsterdam, Netherlands).

[186] Shi CJ, Pang FX, Lei YH, Deng LQ, Pan FZ, Liang ZQ (2025). 5-methylcytosine methylation of MALAT1 promotes resistance to sorafenib in hepatocellular carcinoma through ELAVL1/SLC7A11-mediated ferroptosis. Drug resistance updates: reviews and commentaries in antimicrobial and anticancer chemotherapy.

[187] Li O, An K, Wang H, Li X, Wang Y, Huang L (2025). Targeting YBX1-m5C mediates RNF115 mRNA circularisation and translation to enhance vulnerability of ferroptosis in hepatocellular carcinoma. Clinical and translational medicine.

[188] Chen Y, Jiang Z, Zhang C, Zhang L, Chen H, Xiao N (2024). 5-Methylcytosine transferase NSUN2 drives NRF2-mediated ferroptosis resistance in non-small cell lung cancer. The Journal of biological chemistry.

[189] He M, Wang Y, Xie J, Pu J, Shen Z, Wang A (2024). M(7)G modification of FTH1 and pri-miR-26a regulates ferroptosis and chemotherapy resistance in osteosarcoma. Oncogene.

[190] Porporato PE, Filigheddu N, Pedro JMB, Kroemer G, Galluzzi L (2018). Mitochondrial metabolism and cancer. Cell research.

[191] Zhuang C, Zhuang C, Luo X, Huang X, Yao L, Li J (2019). N6-methyladenosine demethylase FTO suppresses clear cell renal cell carcinoma through a novel FTO-PGC-1α signalling axis. Journal of cellular and molecular medicine.

[192] Sun L, Wan A, Zhou Z, Chen D, Liang H, Liu C (2021). RNA-binding protein RALY reprogrammes mitochondrial metabolism via mediating miRNA processing in colorectal cancer. Gut.

[193] Sun K, Chen L, Li Y, Huang B, Yan Q, Wu C (2023). METTL14-dependent maturation of pri-miR-17 regulates mitochondrial homeostasis and induces chemoresistance in colorectal cancer. Cell death & disease.

[194] Zhou Y, Wang Q, Deng H, Xu B, Zhou Y, Liu J (2022). N6-methyladenosine demethylase FTO promotes growth and metastasis of gastric cancer via m(6)A modification of caveolin-1 and metabolic regulation of mitochondrial dynamics. Cell death & disease.

[195] Jin Y, Qiu J, Lu X, Ma Y, Li G (2023). LncRNA CACNA1G-AS1 up-regulates FTH1 to inhibit ferroptosis and promote malignant phenotypes in ovarian cancer cells. Oncology research.

[196] Wang J, Zheng F, Wang D, Yang Q (2024). Regulation of ULK1 by WTAP/IGF2BP3 axis enhances mitophagy and progression in epithelial ovarian cancer. Cell death & disease.

[197] Sun Y, Shen W, Hu S, Lyu Q, Wang Q, Wei T (2023). METTL3 promotes chemoresistance in small cell lung cancer by inducing mitophagy. Journal of experimental & clinical cancer research: CR.

[198] Wu J, Zhao Q, Chen S, Xu H, Zhang R, Cai D (2024). NSUN4-mediated m5C modification of circERI3 promotes lung cancer development by altering mitochondrial energy metabolism. Cancer letters.

[199] Cai D, Chen X, Xu H, Zhao Q, Zhou X, Wu J (2025). m5C-modified circRREB1 promotes lung cancer progression by inducing mitophagy. Journal of experimental & clinical cancer research: CR.

[200] Chen Z, Zeng C, Yang L, Che Y, Chen M, Sau L (2025). YTHDF2 promotes ATP synthesis and immune evasion in B cell malignancies. Cell.

[201] Luo W, Xu Z, Li F, Ding L, Wang R, Lin Y (2024). m6Am Methyltransferase PCIF1 Promotes LPP3 Mediated Phosphatidic Acid Metabolism and Renal Cell Carcinoma Progression. Advanced science (Weinheim, Baden-Wurttemberg, Germany).

[202] Xu X, Huang Z, Han H, Yu Z, Ye L, Zhao Z (2025). N(7)-methylguanosine tRNA modification promotes gastric cancer progression by activating SDHAF4-dependent mitochondrial oxidative phosphorylation. Cancer letters.

[203] Deng Y, Tan Z, Cai S, Feng Y, Tang Z, Li J (2024). N1-methyladenosine RNA methylation patterns are associated with an increased risk to biochemical recurrence in prostate cancer and serve as a potential novel biomarker for patient stratification. International immunopharmacology.

[204] Peng B, Bartkowiak K, Song F, Nissen P, Schlüter H, Siebels B (2024). Hypoxia-Induced Adaptations of N-Glycomes and Proteomes in Breast Cancer Cells and Their Secreted Extracellular Vesicles. International journal of molecular sciences.

[205] Zhang J, Ouyang F, Gao A, Zeng T, Li M, Li H (2024). ESM1 enhances fatty acid synthesis and vascular mimicry in ovarian cancer by utilizing the PKM2-dependent warburg effect within the hypoxic tumor microenvironment. Molecular cancer.

[206] Liu X, Feng M, Hao X, Gao Z, Wu Z, Wang Y (2023). m6A methylation regulates hypoxia-induced pancreatic cancer glycolytic metabolism through ALKBH5-HDAC4-HIF1α positive feedback loop. Oncogene.

[207] Tang R, Zhang Z, Liu X, Liao Y, Chen Y, Xiao M (2025). Stromal Stiffness-Regulated IGF2BP2 in Pancreatic Cancer Drives Immune Evasion via Sphingomyelin Metabolism. Gastroenterology.

[208] Xiao S, Ma S, Sun B, Pu W, Duan S, Han J (2024). The tumor-intrinsic role of the m(6)A reader YTHDF2 in regulating immune evasion. Science immunology.

[209] Wang A, Huang H, Shi JH, Yu X, Ding R, Zhang Y (2023). USP47 inhibits m6A-dependent c-Myc translation to maintain regulatory T cell metabolic and functional homeostasis. The Journal of clinical investigation.

[210] Xiong J, He L, Chai X, Zhang Y, Sun S (2024). YTHDF1 boosts the lactate accumulation to potentiate cervical cancer cells immune escape. Cell death & disease.

[211] Sun M, Yue Y, Wang X, Feng H, Qin Y, Chen M (2024). ALKBH5-mediated upregulation of CPT1A promotes macrophage fatty acid metabolism and M2 macrophage polarization, facilitating malignant progression of colorectal cancer. Experimental cell research.

[212] Wei Y, Li W, Wu R, Cao Y, Yang S (2025). hnRNPA2B1 potentiates the immune escape of non-small cell lung cancer by accelerating tumor microenvironment acidification. Free radical biology & medicine.

[213] Yang X, Sun L, Guo J, Zheng Y, Ren T, Liu Y (2025). Deciphering the role of IGF2BP2 and PRMT5 in gallbladder cancer progression: insights from multi-omics analysis. British journal of cancer.

[214] Huang F, Wang Y, Zhang X, Gao W, Li J, Yang Y (2025). m(6)A/IGF2BP3-driven serine biosynthesis fuels AML stemness and metabolic vulnerability. Nature communications.

[215] Wang CR, Gong JH, Zhao ZB, Zhu Q, Shu B, Hu JJ (2024). m(6)A demethylation of FOSL1 mRNA protects hepatoma cells against necrosis under glucose deprivation. Cell death and differentiation.

[216] Zhao Y, Wen S, Li H, Pan CW, Wei Y, Huang T (2023). Enhancer RNA promotes resistance to radiotherapy in bone-metastatic prostate cancer by m(6)A modification. Theranostics.

[217] Visvanathan A, Patil V, Arora A, Hegde AS, Arivazhagan A, Santosh V (2018). Essential role of METTL3-mediated m(6)A modification in glioma stem-like cells maintenance and radioresistance. Oncogene.

[218] Niu X, Peng L, Liu W, Miao C, Chen X, Chu J (2022). A cis-eQTL in NSUN2 promotes esophageal squamous-cell carcinoma progression and radiochemotherapy resistance by mRNA-m(5)C methylation. Signal transduction and targeted therapy.

[219] Yu M, Ni M, Xu F, Liu C, Chen L, Li J (2024). NSUN6-mediated 5-methylcytosine modification of NDRG1 mRNA promotes radioresistance in cervical cancer. Molecular cancer.

[220] Shi Y, Fan S, Wu M, Zuo Z, Li X, Jiang L (2019). YTHDF1 links hypoxia adaptation and non-small cell lung cancer progression. Nature communications.

[221] Wang Y, Wei J, Feng L, Li O, Huang L, Zhou S (2023). Aberrant m5C hypermethylation mediates intrinsic resistance to gefitinib through NSUN2/YBX1/QSOX1 axis in EGFR-mutant non-small-cell lung cancer. Molecular cancer.

[222] Gao W, Chen L, Lin L, Yang M, Li T, Wei H (2022). SIAH1 reverses chemoresistance in epithelial ovarian cancer via ubiquitination of YBX-1. Oncogenesis.

[223] Meng H, Miao H, Zhang Y, Chen T, Yuan L, Wan Y (2024). YBX1 promotes homologous recombination and resistance to platinum-induced stress in ovarian cancer by recognizing m5C modification. Cancer letters.

[224] Taketo K, Konno M, Asai A, Koseki J, Toratani M, Satoh T (2018). The epitranscriptome m6A writer METTL3 promotes chemo- and radioresistance in pancreatic cancer cells. International journal of oncology.

[225] Sun G, Ma S, Zheng Z, Wang X, Chen S, Chang T (2022). Multi-omics analysis of expression and prognostic value of NSUN members in prostate cancer. Frontiers in oncology.

[226] Tang B, Yang Y, Kang M, Wang Y, Wang Y, Bi Y (2020). m(6)A demethylase ALKBH5 inhibits pancreatic cancer tumorigenesis by decreasing WIF-1 RNA methylation and mediating Wnt signaling. Molecular cancer.

[227] Ma MJ, Shi YH, Liu ZD, Zhu YQ, Zhao GY, Ye JY (2024). N6-methyladenosine modified TGFB2 triggers lipid metabolism reprogramming to confer pancreatic ductal adenocarcinoma gemcitabine resistance. Oncogene.

[228] Liu X, Gonzalez G, Dai X, Miao W, Yuan J, Huang M (2020). Adenylate Kinase 4 Modulates the Resistance of Breast Cancer Cells to Tamoxifen through an m(6)A-Based Epitranscriptomic Mechanism. Molecular therapy: the journal of the American Society of Gene Therapy.

[229] Chen Z, Wu L, Zhou J, Lin X, Peng Y, Ge L (2020). N6-methyladenosine-induced ERRγ triggers chemoresistance of cancer cells through upregulation of ABCB1 and metabolic reprogramming. Theranostics.

[230] Li Y, Jin H, Li Q, Shi L, Mao Y, Zhao L (2024). The role of RNA methylation in tumor immunity and its potential in immunotherapy. Molecular cancer.

[231] Yan Z, He M, He L, Wei L, Zhang Y (2025). RNA m(6)A methylation patterns in hepatocellular carcinoma and their association with characteristics of the tumor microenvironment and prognosis. Discover oncology.

[232] Wang R, Peng Y, Chen R, Liu R, Ye Y, Chen Q (2025). RNA adenosine modification writers define characteristics of immunity and prognosis in head and neck squamous cell carcinoma. Discover oncology.

[233] Wang Y, Mao Y, Wang C, Jiang X, Tang Q, Wang L (2023). RNA methylation-related genes of m6A, m5C, and m1A predict prognosis and immunotherapy response in cervical cancer. Annals of medicine.

[234] Huang ZD, Fu YC, Liu SY, Mao YJ, Zhang Y, Hu C (2022). m6A-related metabolism molecular classification with distinct prognosis and immunotherapy response in soft tissue sarcoma. Frontiers in immunology.

[235] Zhang R, Gan W, Zong J, Hou Y, Zhou M, Yan Z (2023). Developing an m5C regulator-mediated RNA methylation modification signature to predict prognosis and immunotherapy efficacy in rectal cancer. Frontiers in immunology.

[236] Zhang M, Song J, Yuan W, Zhang W, Sun Z (2021). Roles of RNA Methylation on Tumor Immunity and Clinical Implications. Frontiers in immunology.

[237] Ni Z, Sun P, Zheng J, Wu M, Yang C, Cheng M (2022). JNK Signaling Promotes Bladder Cancer Immune Escape by Regulating METTL3-Mediated m6A Modification of PD-L1 mRNA. Cancer research.

[238] Qiao Z, Li Y, Cheng Y, Li S, Liu S (2023). SHMT2 regulates esophageal cancer cell progression and immune Escape by mediating m6A modification of c-myc. Cell & bioscience.

[239] Tang W, Xu N, Zhou J, He Z, Lenahan C, Wang C (2022). ALKBH5 promotes PD-L1-mediated immune escape through m6A modification of ZDHHC3 in glioma. Cell death discovery.

[240] Wang Y, Jin P, Wang X (2023). N(6)-methyladenosine regulator YTHDF1 represses the CD8 + T cell-mediated antitumor immunity and ferroptosis in prostate cancer via m(6)A/PD-L1 manner. Apoptosis: an international journal on programmed cell death.

[241] Wan W, Ao X, Chen Q, Yu Y, Ao L, Xing W (2022). METTL3/IGF2BP3 axis inhibits tumor immune surveillance by upregulating N(6)-methyladenosine modification of PD-L1 mRNA in breast cancer. Molecular cancer.

[242] Wang S, Zhang X, Chen Q, Wu H, Cao S, Zhao S (2025). FTO activates PD-L1 promotes immunosuppression in breast cancer via the m6A/YTHDF3/PDK1 axis under hypoxic conditions. Journal of advanced research.

[243] Hua X, Xu Q, Wu R, Sun W, Gu Y, Zhu S (2024). ALKBH5 promotes non-small cell lung cancer progression and susceptibility to anti-PD-L1 therapy by modulating interactions between tumor and macrophages. Journal of experimental & clinical cancer research: CR.

[244] Tian F, Huang J, Fan W, Li X, Zhan Y, Zhu K (2025). m(6)A-modified EHD1 controls PD-L1 endosomal trafficking to modulate immune evasion and immunotherapy responses in lung adenocarcinoma. Cancer communications (London, England).

[245] Yang Y, Cao L, Xu X, Li D, Deng Y, Li L (2025). NSUN2/ALYREF axis-driven m(5)C methylation enhances PD-L1 expression and facilitates immune evasion in non-small-cell lung cancer. Cancer immunology, immunotherapy: CII.

[246] Li J, Xu X, Xu K, Zhou X, Wu K, Yao Y (2024). N6-methyladenosine-modified circSLCO1B3 promotes intrahepatic cholangiocarcinoma progression via regulating HOXC8 and PD-L1. Journal of experimental & clinical cancer research: CR.

[247] Sun XY, Yu B, Yu J, Wang YP, Li XB, Sun RR (2025). YBX1 is required for maintaining PD-L1 expression in intrahepatic cholangiocarcinoma by regulating STAT1 stability in an m5C-dependent manner. Hepatobiliary & pancreatic diseases international: HBPD INT.

[248] Winkler R, Gillis E, Lasman L, Safra M, Geula S, Soyris C (2019). m(6)A modification controls the innate immune response to infection by targeting type I interferons. Nature immunology.

[249] Rubio RM, Depledge DP, Bianco C, Thompson L, Mohr I (2018). RNA m(6) A modification enzymes shape innate responses to DNA by regulating interferon β. Genes & development.

[250] Yang S, Wei J, Cui YH, Park G, Shah P, Deng Y (2019). m(6)A mRNA demethylase FTO regulates melanoma tumorigenicity and response to anti-PD-1 blockade. Nature communications.

[251] Lu L, Gaffney SG, Cannataro VL, Townsend J (2020). Transfer RNA methyltransferase gene NSUN2 mRNA expression modifies the effect of T cell activation score on patient survival in head and neck squamous carcinoma. Oral oncology.

[252] Tao Z, Ruan H, Sun L, Kuang D, Song Y, Wang Q (2019). Targeting the YB-1/PD-L1 Axis to Enhance Chemotherapy and Antitumor Immunity. Cancer immunology research.

[253] Moroz-Omori EV, Huang D, Kumar Bedi R, Cheriyamkunnel SJ, Bochenkova E, Dolbois A (2021). METTL3 Inhibitors for Epitranscriptomic Modulation of Cellular Processes. ChemMedChem.

[254] Dolbois A, Bedi RK, Bochenkova E, Müller A, Moroz-Omori EV, Huang D (2021). 1,4,9-Triazaspiro[5.5]undecan-2-one Derivatives as Potent and Selective METTL3 Inhibitors. Journal of medicinal chemistry.

[255] Yankova E, Blackaby W, Albertella M, Rak J, De Braekeleer E, Tsagkogeorga G (2021). Small-molecule inhibition of METTL3 as a strategy against myeloid leukaemia. Nature.

[256] Eleftheriou M, Russell J, Tzelepis K (2025). Epitranscriptomic advances in normal and malignant hematopoiesis. Leukemia.

[257] Chen B, Ye F, Yu L, Jia G, Huang X, Zhang X (2012). Development of cell-active N6-methyladenosine RNA demethylase FTO inhibitor. Journal of the American Chemical Society.

[258] Yan F, Al-Kali A, Zhang Z, Liu J, Pang J, Zhao N (2018). A dynamic N(6)-methyladenosine methylome regulates intrinsic and acquired resistance to tyrosine kinase inhibitors. Cell research.

[259] Singh B, Kinne HE, Milligan RD, Washburn LJ, Olsen M, Lucci A (2016). Important Role of FTO in the Survival of Rare Panresistant Triple-Negative Inflammatory Breast Cancer Cells Facing a Severe Metabolic Challenge. PloS one.

[260] Toh JDW, Sun L, Lau LZM, Tan J, Low JJA, Tang CWQ (2015). A strategy based on nucleotide specificity leads to a subfamily-selective and cell-active inhibitor of N(6)-methyladenosine demethylase FTO. Chemical science.

[261] Huang Y, Su R, Sheng Y, Dong L, Dong Z, Xu H (2019). Small-Molecule Targeting of Oncogenic FTO Demethylase in Acute Myeloid Leukemia. Cancer cell.

[262] Su R, Dong L, Li Y, Gao M, Han L, Wunderlich M (2020). Targeting FTO Suppresses Cancer Stem Cell Maintenance and Immune Evasion. Cancer cell.

[263] Su R, Dong L, Li C, Nachtergaele S, Wunderlich M, Qing Y (2018). R-2HG Exhibits Anti-tumor Activity by Targeting FTO/m(6)A/MYC/CEBPA Signaling. Cell.

[264] Huang Y, Yan J, Li Q, Li J, Gong S, Zhou H (2015). Meclofenamic acid selectively inhibits FTO demethylation of m6A over ALKBH5. Nucleic acids research.

[265] Xiao L, Li X, Mu Z, Zhou J, Zhou P, Xie C (2020). FTO Inhibition Enhances the Antitumor Effect of Temozolomide by Targeting MYC-miR-155/23a Cluster-MXI1 Feedback Circuit in Glioma. Cancer research.

[266] Malacrida A, Rivara M, Di Domizio A, Cislaghi G, Miloso M, Zuliani V (2020). 3D proteome-wide scale screening and activity evaluation of a new ALKBH5 inhibitor in U87 glioblastoma cell line. Bioorganic & medicinal chemistry.

[267] Li N, Kang Y, Wang L, Huff S, Tang R, Hui H (2020). ALKBH5 regulates anti-PD-1 therapy response by modulating lactate and suppressive immune cell accumulation in tumor microenvironment. Proceedings of the National Academy of Sciences of the United States of America.

[268] Selberg S, Seli N, Kankuri E, Karelson M (2021). Rational Design of Novel Anticancer Small-Molecule RNA m6A Demethylase ALKBH5 Inhibitors. ACS omega.

[269] Micaelli M, Dalle Vedove A, Cerofolini L, Vigna J, Sighel D, Zaccara S (2022). Small-Molecule Ebselen Binds to YTHDF Proteins Interfering with the Recognition of N (6)-Methyladenosine-Modified RNAs. ACS pharmacology & translational science.

[270] Wang L, Dou X, Chen S, Yu X, Huang X, Zhang L (2023). YTHDF2 inhibition potentiates radiotherapy antitumor efficacy. Cancer cell.

[271] Wallis N, Oberman F, Shurrush K, Germain N, Greenwald G, Gershon T (2022). Small molecule inhibitor of Igf2bp1 represses Kras and a pro-oncogenic phenotype in cancer cells. RNA biology.

[272] Ge L, Zhang N, Chen Z, Song J, Wu Y, Li Z (2020). Level of N6-Methyladenosine in Peripheral Blood RNA: A Novel Predictive Biomarker for Gastric Cancer. Clinical chemistry.

[273] Xie J, Huang Z, Jiang P, Wu R, Jiang H, Luo C (2021). Elevated N6-Methyladenosine RNA Levels in Peripheral Blood Immune Cells: A Novel Predictive Biomarker and Therapeutic Target for Colorectal Cancer. Frontiers in immunology.

[274] Yin H, Huang Z, Niu S, Ming L, Jiang H, Gu L (2022). 5-Methylcytosine (m(5)C) modification in peripheral blood immune cells is a novel non-invasive biomarker for colorectal cancer diagnosis. Frontiers in immunology.

[275] Pei Y, Lou X, Li K, Xu X, Guo Y, Xu D (2020). Peripheral Blood Leukocyte N6-methyladenosine is a Noninvasive Biomarker for Non-small-cell Lung Carcinoma. OncoTargets and therapy.

[276] Huang M, Ming L, Jiang H, Jiang P, Jiang X, Yin H (2023). Diagnostic value of aberrant decreased 5-Methylcytosine RNA modification in leukocytes for non-small cell lung cancer. Journal of Cancer.

[277] Xiao H, Fan X, Zhang R, Wu G (2021). Upregulated N6-Methyladenosine RNA in Peripheral Blood: Potential Diagnostic Biomarker for Breast Cancer. Cancer research and treatment.

[278] Wang S, Chai P, Jia R, Jia R (2018). Novel insights on m(6)A RNA methylation in tumorigenesis: a double-edged sword. Molecular cancer.

[279] Raj N, Wang M, Seoane JA, Zhao RL, Kaiser AM, Moonie NA (2022). The Mettl3 epitranscriptomic writer amplifies p53 stress responses. Molecular cell.

[280] Qiu X, Yang S, Wang S, Wu J, Zheng B, Wang K (2021). M(6)A Demethylase ALKBH5 Regulates PD-L1 Expression and Tumor Immunoenvironment in Intrahepatic Cholangiocarcinoma. Cancer research.

[281] Yang J, Xu J, Wang W, Zhang B, Yu X, Shi S (2023). Epigenetic regulation in the tumor microenvironment: molecular mechanisms and therapeutic targets. Signal transduction and targeted therapy.

[282] An Y, Duan H (2022). The role of m6A RNA methylation in cancer metabolism. Molecular cancer.

[283] Gong T, Rai SK, Zhu Y, Wang Y, Chen Y, Ma L (2025). Integrative epitranscriptomic and transcriptomic characterization in human colorectal cancer. Journal of advanced research.

